# Presynaptic α_2_δ-2 Calcium Channel Subunits Regulate Postsynaptic GABA_A_ Receptor Abundance and Axonal Wiring

**DOI:** 10.1523/JNEUROSCI.2234-18.2019

**Published:** 2019-04-03

**Authors:** Stefanie Geisler, Clemens L. Schöpf, Ruslan Stanika, Marcus Kalb, Marta Campiglio, Daniele Repetto, Larissa Traxler, Markus Missler, Gerald J. Obermair

**Affiliations:** ^1^Division of Physiology, Medical University Innsbruck, 6020 Innsbruck, Austria, and; ^2^Institute of Anatomy and Molecular Neurobiology, Westfälische Wilhelms-University, 48149 Münster, Germany

**Keywords:** auxiliary subunits, cacna2d, cultured hippocampal neurons, imaging, immunocytochemistry, voltage-gated calcium channels

## Abstract

Presynaptic α_2_δ subunits of voltage-gated calcium channels regulate channel abundance and are involved in glutamatergic synapse formation. However, little is known about the specific functions of the individual α_2_δ isoforms and their role in GABAergic synapses. Using primary neuronal cultures of embryonic mice of both sexes, we here report that presynaptic overexpression of α_2_δ-2 in GABAergic synapses strongly increases clustering of postsynaptic GABA_A_Rs. Strikingly, presynaptic α_2_δ-2 exerts the same effect in glutamatergic synapses, leading to a mismatched localization of GABA_A_Rs. This mismatching is caused by an aberrant wiring of glutamatergic presynaptic boutons with GABAergic postsynaptic positions. The trans-synaptic effect of α_2_δ-2 is independent of the prototypical cell-adhesion molecules α-neurexins (α-Nrxns); however, α-Nrxns together with α_2_δ-2 can modulate postsynaptic GABA_A_R abundance. Finally, exclusion of the alternatively spliced exon 23 of α_2_δ-2 is essential for the trans-synaptic mechanism. The novel function of α_2_δ-2 identified here may explain how abnormal α_2_δ subunit expression can cause excitatory–inhibitory imbalance often associated with neuropsychiatric disorders.

**SIGNIFICANCE STATEMENT** Voltage-gated calcium channels regulate important neuronal functions such as synaptic transmission. α_2_δ subunits modulate calcium channels and are emerging as regulators of brain connectivity. However, little is known about how individual α_2_δ subunits contribute to synapse specificity. Here, we show that presynaptic expression of a single α_2_δ variant can modulate synaptic connectivity and the localization of inhibitory postsynaptic receptors. Our findings provide basic insights into the development of specific synaptic connections between nerve cells and contribute to our understanding of normal nerve cell functions. Furthermore, the identified mechanism may explain how an altered expression of calcium channel subunits can result in aberrant neuronal wiring often associated with neuropsychiatric disorders such as autism or schizophrenia.

## Introduction

Auxiliary α_2_δ subunits are traditionally envisioned as potent modulators of voltage-gated calcium channels (Felix et al., 1997; Klugbauer et al., 1999). Recently, synaptic functions of all four α_2_δ subunit isoforms have been suggested, which may be partly or entirely independent of the channel complex: α_2_δ-1 has been identified as postsynaptic receptor for glia-secreted thrombospondins, which mediate excitatory synaptogenesis (Eroglu et al., 2009; Risher et al., 2018). In mice, loss of α_2_δ-2 affected morphology and function of cerebellar Purkinje neurons (Barclay et al., 2001; Brodbeck et al., 2002), as well as structure and function of auditory hair cell synapses (Fell et al., 2016). Additionally, α_2_δ-2 has been identified as a suppressor of axonal regeneration in the adult CNS (Tedeschi et al., 2016). The invertebrate homologs of α_2_δ-3 are crucial for the presynaptic development of motoneurons (Kurshan et al., 2009; Caylor et al., 2013) and, in mice, loss of α_2_δ-3 results in aberrant synapse formation of auditory nerve fibers (Pirone et al., 2014). Finally, α_2_δ-4 is required for the organization of rod and cone photoreceptor synapses (Wang et al., 2017; Kerov et al., 2018). The overall importance of α_2_δ subunits is emphasized by their involvement in various neurological disorders: CACNA2D1 and CACNA2D2, the genes encoding α_2_δ-1 and α_2_δ-2, have been linked to epilepsy (Chioza et al., 2009; Edvardson et al., 2013; Pippucci et al., 2013; Vergult et al., 2015; Butler et al., 2018); CACNA2D3 is a potential risk gene for autism spectrum disorders (Iossifov et al., 2012; De Rubeis et al., 2014); and all three genes may be associated with schizophrenia (Purcell et al., 2014; Moons et al., 2016). Mutations in CACNA2D4 can cause retinal dysfunction in humans (Ba-Abbad et al., 2016) and a partial deletion of CACNA2D4 was identified in patients with late-onset bipolar disorder (Van Den Bossche et al., 2012).

Despite the increasing knowledge, it is still largely elusive how the individual α_2_δ isoforms contribute to the formation and function of central synapses. The fact that α_2_δ subunits constitute large extracellular and highly glycosylated proteins (Davies et al., 2007; Bauer et al., 2010) puts them in an ideal position for extracellular functions, as previously suggested (Fell et al., 2016; Wang et al., 2017). Indeed, a recent study provided evidence for an interaction of α_2_δ-1 with NMDARs in dorsal horn neurons (Chen et al., 2018) and presynaptic α-neurexins (α-Nrxns) are potential candidates for mediating synaptic functions of α_2_δ subunits (Tong et al., 2017; Brockhaus et al., 2018). However, the role of α_2_δ subunits has until now only been addressed in excitatory glutamatergic synapses. This is surprising because α_2_δ subunits are strongly expressed in GABAergic neurons (Cole et al., 2005; Schlick et al., 2010) and aberrant GABAergic signaling is primarily implicated in the etiology of the abovementioned neurological disorders (e.g., epilepsy; Bonansco and Fuenzalida, 2016). Finally, the brain robustly expresses α_2_δ-1, α_2_δ-2, and α_2_δ-3 (Schlick et al., 2010); however, whether and how the individual isoforms contribute to specific functions in neurons simultaneously expressing all isoforms is elusive.

Therefore, to address α_2_δ subunit-mediated synaptic functions, we analyzed the consequences of presynaptic α_2_δ subunit expression in glutamatergic and GABAergic synapses. We show that presynaptic overexpression of α_2_δ-2 specifically induces the formation of mismatched glutamatergic synapses by trans-synaptically recruiting postsynaptic GABA_A_Rs. Moreover, α_2_δ-2 recruits postsynaptic GABA_A_Rs also in GABAergic synapses and thus independently of the presynaptic neurotransmitter identity. Most importantly, the mismatched synapse formation is explained by an altered wiring of glutamatergic axons to GABAergic postsynaptic positions. Interestingly, the α_2_δ-2-induced GABA_A_R clustering is further upregulated in glutamatergic neurons lacking all three α-Nrxns. Therefore, α-Nrxns can modulate the presynaptic effect of α_2_δ-2; however, they are not required for the trans-synaptic role of α_2_δ-2 in recruiting GABA_A_Rs. Together, our findings prove that presynaptic α_2_δ-2 acts trans-synaptically on postsynaptic GABA_A_R. The fact that increased α_2_δ-2 expression triggers aberrant axonal wiring is particularly interesting in light of neurological disorders associated with axonal wiring defects and altered excitatory–inhibitory balance (Huang and Hsueh, 2015; Lee et al., 2017).

## Materials and Methods

### 

#### Breeding and genotyping procedures

##### Animals.

Animal procedures for WT BALB/c and α_2_δ mutant mice were performed at the Medical University Innsbruck in compliance with government regulations and approved by the Austrian Federal Ministry of Science, Research and Economy (license numbers BMWFW-66.011/0113-WF/V/3b/2014 and BMWFW-66.011/0114-WF/V/3b/2014). Regular reports including the mouse numbers used for this project were given to the Austrian Federal Ministry of Science, Research and Economy (bmwfw). Animal experiments at the University of Münster [α-Nrxn triple knock-out (TKO) and control mice] were performed in accordance with government regulations for animal welfare and approved by the Landesamt für Natur, Umwelt und Verbraucherschutz (LANUV, NRW, Germany, license numbers 84-02.05.20.11.209 and 84-02.04.2015.A423). Mice were maintained at the central animal facilities in Innsbruck and Münster under standard housing conditions with food and water available *ad libitum* on a 12 h light/dark cycle.

##### Breeding and genotyping of mutant mice.

WT and α_2_δ-3^−/−^ mice used for qRT-PCR and LacZ reporter expression were obtained from breeding double heterozygous α_2_δ-1^+/−^, α_2_δ-3^+/−^ mice having a mixed 129J × C57BL/6 background. The α_2_δ-1 knock-out mouse strain (α_2_δ-1^−/−^) was previously generated and characterized (Fuller-Bicer et al., 2009; Patel et al., 2013; Mastrolia et al., 2017). Genotyping for the Cacna2d1 gene was done as published previously (Fuller-Bicer et al., 2009) with some modifications by use of standard PCR conditions (annealing at 52°C for 30 s). Primers were as follows: WT-F1: 5′-GAGCTTTCTTTCTTCTGATTCCAC-3′, mutant-F2: 5′-CTGCACGAGACTAGTGAGACG-3′, R: 5′-ACATTCTCAAGACTGTAGGCAGAG-3′. Expected band sizes were 346 bp for WT (α_2_δ-1^+/+^) and 635 bp for knock-out (α_2_δ-1^−/−^) animals, respectively, and heterozygous mice showed both bands. The α_2_δ-3 knock-out mice (α_2_δ-3^−/−^) generated by Deltagen (B6.129P2-Cacna2d3^tm1Dgen^; Neely et al., 2010) were originally purchased from The Jackson Laboratory and provided by Jutta Engel (Saarland University, Germany). Knock-out was obtained by targeted insertion of a bacterial LacZ cassette into the Cacna2d3 gene such that the endogenous promoter drives the expression of β-galactosidase. The following primers were used for detecting the WT allele (F1-R, 183 bp fragment) and the knock-out allele (F2-R, 331 bp fragment): F1: 5′-TAGAAAAGATGCACTGGTCACCAGG-3′, F2: 5′-GGGCCAGCTCATTCCTCCCACTCAT-3′, R: 5′-GCAGAAGGCACATTGCCATACTCAC-3′ by use of standard PCR conditions (annealing at 63°C for 30 s). α-Nrxn TKO mice were generated as described previously (Missler et al., 2003; Dudanova et al., 2007). Genotyping of α-Nrxn-deficient mice was performed at the same time of neuronal preparation and as described previously (Missler et al., 2003).

#### Cell culture and transfection procedures

##### Lentiviral production.

Lentiviruses were produced by transient transfection of confluent tsa201 cells with the lentiviral expression vectors containing pHR-βA-eGFP, pHR-βA-mcherry, or pHR-βA-eGFP*α_2_δ-2 in combination with psPAX2 (packaging plasmid) and the pVSV (envelope plamid) using Metafectene (Biontex Laboratories). The following day, medium was changed to neuronal plating medium (NPM; consisting of MEM, 10% horse serum, 0.2% glucose, and 1 mm sodium pyruvate) and, after 24 and 48 h, supernatants containing the viruses were harvested, sterile filtered (0.20 μm), aliquoted, and stored at −20°C. Cultured hippocampal and striatal neurons were infected immediately after plating with the lentiviral medium supernatant diluted 1:4 in NPM and incubated for 4 h in a humidified incubator (95% air and 5% CO_2_) at 37°C. More details for infection procedures are given in the individual sections below.

##### Primary cultures of hippocampal neurons for fluorescence imaging.

Low-density cultures of hippocampal neurons were obtained from 16.5- to 18-d-old embryonic BALB/c mice of either sex as described previously (Obermair et al., 2004; Kaech and Banker, 2006; Stanika et al., 2016; Folci et al., 2018). In brief, hippocampi were dissected in cold Hank's balanced salt solution (HBSS) following dissociation by 2.5% trypsin-EDTA treatment and trituration. Dissociated neurons were plated at a density of ∼3500 or 7000 cells/cm^2^ on 18 mm glass coverslips (#1.5; GML) coated with poly-l-lysine (Sigma-Aldrich) in 60 mm culture dishes. After allowing the neurons to attach for 3–4 h, coverslips were transferred neuron-side down into a 60 mm culture dish containing a glial feeder layer. Maintenance of neurons and glia was done in serum-free neurobasal medium supplemented with Glutamax and B-27 (NBKO, all ingredients from Thermo Fisher Scientific). Three days after plating, Ara-C (5 μm) was added to stop glial proliferation and 1/3 of the medium was replaced weekly with fresh maintenance medium. Plasmids were introduced into neurons at 6 days *in vitro* (DIV) with Lipofectamine 2000-mediated transfection (Thermo Fisher Scientific) as described previously (Obermair et al., 2004). For cotransfection of pβA-eGFP plus pβA-α_2_δ or mCherry plus pβA-α_2_δ, 1.5 μg of total DNA was used at a molar ratio of 0.7:1. Control neurons from the same culture preparations were transfected with 1–2 μg of pβA-eGFP or mCherry, respectively. Labeling of cells for superresolution gated stimulated emission depletion (gSTED) microscopy (see Fig. 11*A–C*) was done by introducing mCherry or mCherry plus α_2_δ-2 at 6 DIV and pβA-eGFP at 7 DIV with lipofection. Cells were processed for immunostaining experiments between 20 and 30 DIV.

For α-Nrxn TKO, WT and mutant mice of either sex were used for neuronal cultures derived from timed pregnant dams at embryonic day 17 (E17). Dissociated primary neurons were prepared in HBSS from single hippocampi as described previously (Neupert et al., 2015). Briefly, cell suspensions obtained after 0.25% trypsin treatment and trituration were plated onto 18 mm glass coverslips (Menzel-Glaeser) coated with poly-l-lysine (Sigma-Aldrich) at a density of 55,000 cells/coverslip. After 4 h at 37°C in plating medium (MEM, 10% horse serum, 0.6% glucose, and 1 mm sodium pyruvate), coverslips were inverted onto a 70–80% confluent monolayer of astrocytes grown in 12-well plates (Falcon) and incubated in Neurobasal medium supplemented with B27, 0.5 mm glutamine, and 12.5 μm glutamate. After 3 d, media were refreshed with Neurobasal medium supplemented with B27, 0.5 mm glutamine, and 5 μm AraC. Cultures were maintained at 37°C in a humidified incubator with an atmosphere of 95% air and 5% CO_2_. Neurons were transfected at 14 DIV using lipofectamine (Thermo Fisher Scientific) and experiments were performed between 20 and 25 DIV.

##### Primary cocultures of WT cortical and striatal neurons.

Because the inclusion of glutamatergic neurons is required for the proper development of GABAergic medium spiny neurons (MSNs) in culture (Segal et al., 2003), we modified the protocol for hippocampal neurons and adapted the striatal–cortical coculture published previously (Penrod et al., 2011). Cocultures of cortical neurons and striatal MSNs were prepared from 16.5- to 18-d-old embryonic BALB/c mice of either sex as summarized in Figure 7. Briefly, fetuses were removed from the uterus, decapitated, and brains were dissected in cold HBSS. After separating the cerebral hemispheres and stripping away the meninges, hemispheres were placed medial surface up, showing the hippocampus as a clearly visible structure. A region of the prefrontal cortex was dissected, minced, and transferred to a 15 ml Falcon tube containing HBSS (see Fig. 7*A*). The remaining cortical tissue was peeled along the line of the hippocampus to reveal the striatum, which was scooped out using small curved scissors and transferred to a separate tube. Striatal and cortical tissue of at least 2 hemispheres was separately collected and dissociated by 2.5% trypsin-EDTA treatment and trituration as described for hippocampal neurons (see above). Plasmid DNA–lipid complexes were prepared according to the Lipofectamine 2000-mediated transfection protocol (Thermo Fisher Scientific) and ∼2.4 × 10^5^ striatal neurons were transfected for 20 min in a 37°C water bath keeping the total volume to 1 ml with NBKO. Subsequently, the cell suspension was directly seeded on poly-l-lysine-coated glass coverslips within a 60 mm culture dish containing 4 ml of prewarmed NPM and striatal neurons were allowed to attach at 37°C. For the entire transfection procedure, dissociated cortical neurons were maintained in HBSS in a 15 ml tube at 37°C and occasionally swirled. After 2 h, transfection of striatal neurons was stopped by replacing the transfection-plating solution with 5 ml of fresh, prewarmed NPM and untransfected cortical neurons were seeded onto striatal neurons in a ratio of 2 (cortical neurons) to 3 (MSNs) at a total density of ∼14,000 cells/cm^2^. For the immunostaining experiments and electrophysiological recordings shown in Figure 7, striatal neurons were plated at a density of 4000 or 7000 cells/cm^2^, respectively, and infected with a lentiviral pHR-βA-eGFP construct. Following attachment of MSNs and viral infection for 4 h, untransfected cortical neurons were plated at a density of 2800 cells/cm^2^. Cortical cells were allowed to attach for 3–4 h and coverslips were transferred neuron-side down into a 60 mm culture dish containing a glial feeder layer. In cocultures for superresolution gSTED microscopy (see Fig. 11*E*), striatal and cortical neurons were labeled with eGFP and mCherry, respectively. For this purpose, striatal neurons were plated at a density of 7000 cells/cm^2^ on poly-l-lysine-coated glass coverslips in a 60 mm culture dish containing 4 ml of prewarmed NPM. MSNs were virally infected using a lentiviral pHR-βA-eGFP construct and allowed to attach for 3–4 h. In the meantime, ∼2.0 × 10^5^ cortical neurons were transfected at 37°C in a water bath, keeping the total volume to 1 ml with NBKO. To this end, mCherry or mCherry plus α_2_δ-2 were introduced using the Lipofectamine 2000-mediated transfection protocol (Thermo Fisher Scientific). Total DNA amounts and molar ratios were the same as described above for hippocampal neurons. Infection of striatal neurons was stopped by replacing the virus-plating solution with 4 ml of fresh, prewarmed NPM. The cell suspension containing the cortical neurons and transfection solution was centrifuged for 5 min at 1000 rpm at 4°C. Finally, cortical neurons were resuspended in 1 ml of NBKO, seeded onto striatal neurons, and allowed to attach for 3–4 h before coverslips were transferred neuron-side down into a 60 mm culture dish containing a glial feeder layer. Ara-C treatment and maintenance of neurons and glia were as described above. Cells were processed for immunostaining and patch-clamp experiments at 21–27 and 13–14 DIV, respectively.

##### Neuronal cultures for paired recordings.

Culture protocols were modified to enable electrophysiological recordings from pairs of isolated neurons. Poly-l-lysine solution (Sigma-Aldrich) was sprayed on glass coverslips, producing uniformly distributed single dots of neuronal substrate. Hippocampal neurons were seeded at a density of 880 cells/cm^2^ and infected with pHR-βA-eGFP*α_2_δ-2. Cells were processed for electrophysiological experiments at 13–18 DIV.

#### Molecular biology

##### TaqMan qRT-PCR gene expression analysis.

For expression analysis of α_2_δ subunits (α_2_δ-1 to α_2_δ-4), RNA was isolated from 3 individual preparations of mono-cultured MSNs and adult male mouse striatum (7–8 weeks) as described previously (Schlick et al., 2010). Briefly, striatal neurons were prepared from 16.5- to 18-d-old embryonic BALB/c mice of either sex and plated on poly-l-lysine-coated glass coverslips in four 60 mm culture dishes at a density of 10,500 cells/cm^2^. After 24–25 d in culture, neurons were harvested for subsequent RNA extraction by trypsin treatment and homogenized with QiaShredder columns (Qiagen). For RNA isolation from brain tissue, WT mice obtained from double-heterozygous α_2_δ-1^+/−^, α_2_δ-3^+/−^ breedings were killed by CO_2_ exposure, decapitated, and the striatum was dissected in cold HBSS and stored at −80°C in RNAlater RNA Stabilization Reagent (Qiagen) until further use. Tissue samples were disrupted using a Sonicator (UP200S; Hieschler) and QiaShredder columns (Qiagen). After homogenization, total RNA was immediately isolated with the RNeasy Protect Mini Kit following the manufacturer's instructions (Qiagen) and concentrations were measured using a NanoDrop 2000 spectrophotometer (Thermo Fisher Scientific). Subsequently, 1 μg (striatum) or 5.7 μl (∼50 ng, neurons) of RNA was transcribed with Superscript II reverse transcriptase (Thermo Fisher Scientific) and random hexamers (Promega). qRT-PCR (50 cycles) was performed in duplicates using a maximum amount of 20 ng RNA equivalent of cDNA per sample and the following TaqMan gene expression assays (Thermo Fisher Scientific): α_2_δ-1 (ID: Mm00486607_m1), α_2_δ-2 (ID: Mm00457825_m1), α_2_δ-3 (ID: Mm00486613_m1), α_2_δ-4 (ID: Mm01190105_m1), and Hprt1 (ID: Mm00446968_m1) as an endogenous control. The absolute number of α_2_δ transcripts was calculated by applying standard curves generated from PCR products of known concentrations (Schlick et al., 2010). To compare the relative expression of distinct α_2_δ subunits in different preparations, data were normalized to the preparation with the highest Hprt1 expression.

##### Expression vectors and cloning procedures.

To facilitate neuronal expression, all constructs were cloned into a eukaryotic expression plasmid containing a neuronal chicken β-actin promoter, pβA. Cloning of all constructs was confirmed by sequencing (Eurofins Genomics).

For pβA-α_2_δ-1: Mouse α_2_δ-1 was cloned from genomic cDNA derived from mouse cerebellum. Primer sequences were selected according to GenBank NM-001110844. Briefly, the cDNA of α_2_δ-1 was amplified by PCR in three fragments. The forward primer used for amplifying fragment 1 introduced a NotI site and the Kozak sequence (CCTACC) upstream of the starting codon and the reverse primer used for amplifying fragment 3 introduced a KpnI and a SalI site after the stop codon. Fragment 2 (nt 1442–2564) was MfeI/BamHI digested and fragment 3 (nt 2335–3276) was KpnI/BamHI digested and co-ligated in the corresponding MfeI/KpnI sites of the pβA vector, yielding an intermediate construct. Fragment 1 (nt 1–1575) was NotI/MfeI digested and co-ligated with the SalI/MfeI-digested intermediate construct containing fragments 2 and 3 and the NotI/SalI-digested pβA vector, yielding pβA-α_2_δ-1 (GenBank accession number MK327276).

For pβA-2HA-α_2_δ-1: The putative signal peptide (aa1–24) was predicted using Signal P (SignalP 4.0: discriminating signal peptides from transmembrane regions (Petersen et al., 2011). A double hemagglutinin tag (2HA) followed by a TEV cleavage site was introduced between the third and fourth amino acids after the predicted signal peptide cleavage site of mouse α_2_δ-1; that is, residue F27. Introduction of this sequence did not alter the predicted cleavage site. Briefly, the cDNA sequence of α_2_δ-1 (nt 1–516) was PCR amplified with overlapping primers introducing the double HA tag and the TEV cleavage site in separate PCRs using pβA-α_2_δ-1 as a template. The two separate PCR products were then used as templates for a final PCR with flanking primers to connect the nucleotide sequences. The resulting fragment was then NotI/BglII digested and ligated into the corresponding sites of pβA-α_2_δ-1, yielding pβA-2HA-α_2_δ-1.

For pβA-α_2_δ-2 (v1): Mouse α_2_δ-2 was cloned from genomic cDNA from mouse brain. Primer sequences were selected according to GenBank NM-001174049. The cDNA of α_2_δ-2 was amplified by PCR in four fragments. The forward primer used for amplifying fragment 1 introduced a HindIII site and the Kozak sequence (CCTACC). Fragment 1 was isolated from cerebellum, whereas the other three fragments were isolated from hippocampus. Fragment 1 (nt 1–686) and Fragment 2 (nt 323–1294) were HindIII/BamHI and BamHI/EcoRI digested, respectively, and co-ligated in the corresponding HindIII/EcoRI sites of the pBS (Bluescript) vector, yielding the intermediate construct pBS-α_2_δ-2-part1. Fragment 3 (nt 1137–2359) was EcoRI/PmlI digested and ligated into the corresponding sites of the pSPORT vector, yielding the intermediate construct pSPORT-α_2_δ-2-part2. Fragment 4 (nt 2226–3444) was BmtI/XbaI digested and ligated into the corresponding sites of pSPORT-part2, yielding the intermediate construct pSPORT-α_2_δ-2-part3. pSPORT-α_2_δ-2-part3 (v1) was EcoRI/XbaI digested and the band containing fragments 3–4 (bp 1137–3444) was ligated into pBS-α_2_δ-2-part1, yielding pBS-α_2_δ-2. This construct was then HindIII/XbaI digested and the cDNA of α_2_δ-2 was ligated into the pβA vector, yielding pβA-α_2_δ-2 (v1) (GenBank accession number MK327277).

For pβA-2HA-α_2_δ-2 (v1): A putative signal peptide was not reliably predicted using Signal P (SignalP 4.0: discriminating signal peptides from transmembrane regions; Petersen et al., 2011); however, the highest prediction showed that the signal peptide comprises residues 1–64. The 2HA tag followed by a thrombin cleavage site was therefore introduced after the predicted signal peptide cleavage site of mouse α_2_δ-2; that is, residue A64. Introduction of this sequence did not alter the predicted cleavage site. Briefly the cDNA sequence of α_2_δ-2 (nt 1–761) was PCR amplified with overlapping primers introducing the double HA tag and the thrombin cleavage site in separate PCRs using pβA-α_2_δ-2 as a template. The two separate PCR products were then used as templates for a final PCR with flanking primers to connect the nucleotide sequences. The resulting fragment was then HindIII/AflII digested and ligated into α_2_δ-2 (v1).

For pβA-α_2_δ-2 (v2): An alternative splice variant was isolated from cerebellum, corresponding to GenBank NM-020263. Fragment 3 contained an additional 21 nt at position nt 1992 and was cloned into pSPORT together with fragment 4, yielding pSPORT-α_2_δ-2-part3 (v2). pSPORT-α_2_δ-2-part3 (v2) was digested with ClaI/BglII and cloned into the corresponding sites of pβA-α_2_δ-2 (v1), yielding pβA-α_2_δ-2 (v2) (GenBank accession number MK327278).

For pβA-α_2_δ-2 (v3): An alternative splice variant was isolated from hippocampus, corresponding to GenBank NM-001174048. Fragment 4 contained an additional 3 nt at position nt 2598 (resulting in an additional Q residue) and an additional 6 nt at position 3219 nt (translated in CPA instead of S) and was cloned into pSPORT together with fragment 3, yielding pSPORT-α_2_δ-2-part3 (v3). pSPORT-α_2_δ-2-part3 (v3) was digested with ClaI/XbaI and cloned into the corresponding sites of pβA-α_2_δ-2 (v1), yielding pβA-α_2_δ-2 (v3) (GenBank accession number MK327279).

For pβA-α_2_δ-3: Mouse α_2_δ-3 was cloned from genomic cDNA from mouse hippocampus. Primer sequences were selected according to GenBank NM-009785. Briefly, the cDNA of α_2_δ-3 was amplified by PCR in four fragments. The forward primer used for amplifying fragment 1 introduced a NotI site and the Kozak sequence (CCTACC) upstream of the starting codon. Fragment 3 (nt 1520–2817) was then SacI/PstI digested and fragment 4 (nt 2727–3276) was DraI/PstI digested and co-ligated in the corresponding SacI/SmaI sites of the pSPORT vector, yielding an intermediate construct. Fragment1 (nt 1–653) was then NotI/BamHI digested and fragment2 (535–1636) was SacI/BamHI digested and co-ligated with the SacI/NotI digested intermediate construct containing fragments 3 and 4, yielding pSPORT-α_2_δ-3. The cloned cDNA of α_2_δ-3 was then NotI/RsrII digested and ligated into the corresponding sites of the pβA vector, yielding pβA-α_2_δ-3 (GenBank accession number MK327280).

For pβA-2HA-α_2_δ-3: The putative signal peptide (aa 1–28) was predicted using Signal P (SignalP 4.0: discriminating signal peptides from transmembrane regions; Petersen et al., 2011). The 2HA tag followed by a thrombin cleavage site was therefore introduced after the predicted signal peptide cleavage site of mouse α_2_δ-3; that is, residue D28. Introduction of this sequence did not alter the predicted cleavage site. Briefly, the cDNA sequence of α_2_δ-3 (nt 1–653) was PCR amplified with overlapping primers introducing the double HA tag and the thrombin cleavage site in separate PCRs using pβA-α_2_δ-3 as a template. The two separate PCR products were then used as templates for a final PCR with flanking primers to connect the nucleotide sequences. The resulting fragment was then NotI/BsrGI digested and ligated into the corresponding sites of pβA-α_2_δ-3, yielding pβA-2HA-α_2_δ-3.

For pHR-pβA-eGFP*α_2_δ-2 (v1): To remove the HA tag from pβA-eGFP*2HA-α_2_δ-2, pβA-eGFP*2HA-α_2_δ-2 was XhoI/RsrII digested to isolate part of the promoter and the eGFP coding sequence. The obtained fragment was then inserted in the corresponding sites of pβA-α_2_δ-2, yielding pβA-eGFP*α_2_δ-2. For generating the viral vector pHR-pβA-eGFP*α_2_δ-2, the eGFP*α_2_δ-2 coding sequence was introduced with HindIII/XbaI into a custom-built pENTR vector and inserted into a custom-built destination vector, pHR-βA-DEST, using the LR Clonase II enzyme mixture (GATEWAY; Invitrogen), yielding pHR-pβA-eGFP*α_2_δ-2 (v1).

For pβA-eGFP*2HA-α_2_δ-2 (v1): The eGFP coding sequence, followed by a stop codon and the Kozak sequence (CCTACC), were introduced by PCR between the Kozak sequence and the coding sequence of 2HA-α_2_δ-2. The resulting fragment was then HindIII/PpuMI digested and ligated into the corresponding sites of pβA-2HA-α_2_δ-2, yielding pβA-eGFP*2HA-α_2_δ-2. This construct was then transfected in hippocampal cultures with lipofectamine to insure that both the eGFP and HA signals were present in transfected neurons.

For pHR-pβA-eGFP: The cloning procedure to generate this plasmid was described previously (Subramanyam et al., 2009).

For pHR-pβA-mcherry: The coding sequence of mcherry was inserted into a custom-built destination vector, pHR-βA-DEST, using LR Clonase II enzyme mixture (GATEWAY; Invitrogen) as described previously (Etemad et al., 2014).

#### Immunocytochemistry and microscopy

##### Antibodies.

Information on primary and secondary antibodies is summarized in Table 1.

**Table 1.** List of primary and secondary antibodies

	Species	Dilution	Source
**Primary antibodies**			
Anti-HA	Rat, monoclonal, clone 3F10	1:100 (LIVE/A594)	Roche (catalog #11867423001, RRID:AB_390918)
Anti-GABA_A_R_β2/3_	Mouse, monoclonal, clone bd17	1:500 (A594)	Millipore (catalog #MAB341, RRID:AB_2109419)
		1:250 (A350/OG488)	
Anti-GABA_A_R_α1N_	Rabbit, polyclonal	1:2000 (A594)	(Hörtnagl et al., 2013)
Anti-GABA_A_R_α2C_	Rabbit, polyclonal	1:1000 (A594)	(Hörtnagl et al., 2013)
Anti-GABA_A_R_α3N_	Rabbit, polyclonal	1:500 (A594)	(Hörtnagl et al., 2013)
Anti-GABA_A_R_α4N_	Rabbit, polyclonal	1:500 (A594)	(Hörtnagl et al., 2013)
Anti-GABA_A_R_β2L_	Rabbit, polyclonal	1:500 (A594)	(Hörtnagl et al., 2013)
Anti-GABA_A_R_β3L_	Rabbit, polyclonal	1:2000 (A594)	(Hörtnagl et al., 2013)
Anti-GABA_A_R_γ2L_	Rabbit, polyclonal	1:500 (A594)	(Hörtnagl et al., 2013)
Anti-GABA_A_R_δN_	Rabbit, polyclonal	1:100 (A594)	(Hörtnagl et al., 2013)
Anti-gephyrin	Mouse, monoclonal, clone mab7a	1:2000 (A594)	Synaptic Systems (catalog #147 021, RRID:AB_2232546)
		1:1000 (A350/STAR440)	
Anti-GLUR1	Rabbit, polyclonal	1:1000 (A594)	Upstate (catalog #06-306)
Anti-PSD-95	Mouse, monoclonal, clone 6G6–1C9	1:1000 (A594)	Thermo Fisher Scientific (catalog #MA1-045, RRID:AB_325399)
Anti-synapsin1/2	Rabbit, polyclonal	1:2000 (A350)	Synaptic Systems (catalog #106 002, RRID:AB_887804)
Anti-synapsin1	Mouse, monoclonal, clone 46.1	1:500 (A350)	Synaptic Systems (catalog #106 011, RRID:AB_2619772)
Anti-vGLUT1	Rabbit, polyclonal	1:2000 (A350)	Synaptic Systems (catalog #135 002, RRID:AB_2315546)
Anti-vGLUT1	Mouse, monoclonal, clone 317G6	1:500 (A594)	Synaptic Systems (catalog #135 511, RRID:AB_887879)
		1:250 (A350)	
Anti-vGAT	Rabbit, polyclonal	1:500 (A350)	Synaptic Systems (catalog #131 002, RRID:AB_887871)
		1:250 (STAR440)	
**Secondary antibodies**			
Alexa Fluor 350	Goat anti-mouse	1:500	Thermo Fisher Scientific (catalog #A-21049, RRID:AB_2535717)
	Goat anti-rabbit	1:500	Thermo Fisher Scientific (catalog #A-21068, RRID:AB_2535729)
Alexa Fluor 594	Goat anti-mouse	1:4000	Thermo Fisher Scientific (catalog #A-11032, RRID:AB_2534091)
	Goat anti-rabbit	1:4000	Thermo Fisher Scientific (catalog #A-11037, RRID:AB_2534095)
	Goat anti-rat	1:4000	Thermo Fisher Scientific (catalog #A-11007, RRID:AB_10561522)
Oregon Green 488	Goat anti-mouse	1:500	Thermo Fisher Scientific (catalog #O-11033, RRID:AB_2539797)
STAR 440	Goat anti-mouse	1:500	Abberior (catalog #2-0002-003-7, RRID:AB_2756514)

##### High-resolution fluorescence microscopy.

Immunolabeling of permeabilized or live-cell-stained neurons was performed as described previously (Obermair et al., 2004, 2010; Stanika et al., 2016; Folci et al., 2018) and information on antibodies is given in Table 1. For PSD-95 labeling of eGFP-transfected neurons, cultures were fixed in 4% paraformaldehyde/4% sucrose (pF) in PBS for 20 min at room temperature, rinsed in PBS followed by a second fixation with absolute methanol for 10 min at −20°C. For all other antibody combinations, neurons were fixed with pF only. Subsequent to fixation and washing, cultures were incubated for 30 min in 5% normal goat serum in PBS containing 0.2% bovine serum albumin (BSA) and 0.2% Triton X-100 (PBS/BSA/Triton), enabling membrane permeabilization. Primary antibodies were applied overnight in PBS/BSA/Triton at 4°C and detected by fluorochrome-conjugated secondary antibodies incubated for 1 h at room temperature. For surface staining of HA-tagged α_2_δ constructs, transfected neurons were incubated with rat-anti-HA antibody for 20 min at 37°C following rinsing in warm HBSS and fixation with pF for 10 min at room temperature. Subsequent washing and blocking steps were done with PBS and PBS/BSA, respectively, followed by 1 h incubation with fluorochrome-conjugated secondary goat-anti-rat Alexa Fluor 594 antibody. After rinsing and postfixation of cells in pF for 5 min, neurons were permeabilized by blocking with PBS/BSA/Triton. Primary mouse-anti-synapsin antibody was applied overnight at 4°C and detected with goat-anti-mouse Alexa Fluor 350 antibody. Coverslips were either mounted in p-phenylenediamine glycerol (Flucher et al., 1993) or DAKO fluorescence mounting medium (Agilent Technologies) for α-Nrxn knock-out and control cultures. Hippocampal cultures were mainly viewed with an Axio Imager microscope (Carl Zeiss) using a 63× 1.4 numerical aperture (NA) oil-immersion objective lens and 14-bit grayscale images were recorded with a cooled CCD camera (SPOT; Diagnostic Instruments) using MetaMorph software (Molecular Devices). α-Nrxn knock-out cultures and MSNs were observed with a BX53 microscope (Olympus) using a 60× 1.42 NA oil-immersion objective lens and 14-bit grayscale images were recorded with a cooled CCD camera (XM10; Olympus) using cellSens Dimension software (Olympus). To analyze presynaptic and postsynaptic differentiation cover glasses were systematically screened for contact sites between axons of presynaptic neurons [transfected with the fluorescent marker alone (eGFP or mCherry only) or cotransfected with the marker and target construct (eGFP or mCherry plus α_2_δ)] and dendrites or somas of nontransfected postsynaptic neurons. Images of randomly selected well differentiated cells were acquired with the same exposure and gain settings for all conditions within an experiment. These settings were initially adjusted to facilitate optimal visualization of peripheral cell compartments (axons and presynaptic boutons) also in weakly expressing neurons. Therefore, overly saturated neurons (based on eGFP and mCherry levels) were excluded from analysis and only cells with medium to low eGFP or mCherry expression were selected for further analysis. Images were analyzed with MetaMorph software (Molecular Devices) as described below. Figures were assembled in Adobe Photoshop CS6 and linear adjustments were done to correct black level and contrast.

##### gSTED microscopy.

For gSTED microscopy of mCherry-transfected permeabilized hippocampal and striatal MSNs (see Fig. 3), immunolabeling was done as described for high-resolution fluorescence microscopy with some modifications. Briefly, mouse-anti-GABA_A_R_β2/3_ antibody was used at higher concentrations (1:250) and detected with secondary goat-anti-mouse Oregon Green (OG) 488 (1:500). Coverslips were mounted in DABCO/glycerol to retard photobleaching (2% DABCO, 90% glycerol, buffered in Tris-HCl, pH 8.5; DABCO and glycerol were purchased from Carl Roth). Samples were visualized using a TCS SP8 gSTED microscope equipped with an HCXPL APO 100× 1.4 NA oil-immersion objective (Leica Microsystems) and images were scanned at a pixel size of 15 nm. For hippocampal neurons, individual fluorophores were recorded in the following sequence (excitation/detection range wavelength in nm): mCherry (575/600–690; gate 0.20–6 ns, in confocal mode) and OG 488 (mouse-anti-GABA_A_R_β2/3_; 502/512–581; gate 1–6 ns, STED laser at 592 nm 100%). For MSNs, individual fluorophores were recorded in the following sequence (excitation/detection range wavelength in nm): mCherry (mCherry only or mCherry + α_2_δ-2; 575/600–690; gate 0.20–6 ns, in confocal mode) and OG 488 (mouse-anti-GABA_A_R_β2/3_; 502/510–582; gate 1–6.2 ns, STED laser at 592 nm 100%). To study the localization of excitatory synapses on postsynaptic dendrites of excitatory neurons (see Fig. 11*B*), hippocampal neurons labeled with mCherry and eGFP, respectively, were fixed in 4% pF in PBS for 20 min at room temperature, washed in PBS, and mounted in DABCO/glycerol. Fluorophores were recorded in the following sequence (excitation/detection range wavelength in nm): mCherry (presynaptic axon: mCherry only or mCherry + α_2_δ-2; 584/600–688; gate 0.20–6 ns in confocal mode) and eGFP (postsynaptic dendrite: eGFP only, 490/508–584 or 520–584; gate 1–6.2 ns, STED laser at 592 nm). To enable clear visualization of dendritic structures, STED laser intensity was adjusted according to the eGFP signal. To study the localization of excitatory synapses on postsynaptic dendrites of inhibitory GABAergic neurons (see Fig. 11*E*), cortical neurons and MSNs were labeled with mCherry and eGFP, respectively, as described above. Primary mouse-anti-gephyrin (1:1000) was applied overnight in PBS/BSA/Triton at 4°C and detected with secondary goat-anti-mouse STAR440 (1:500). Fluorophores were recorded in the following sequence (excitation/detection range wavelength in nm): mCherry (presynaptic axon: mCherry only or mCherry + α_2_δ-2; 580/600–690; gate 0.5–6 ns in confocal mode), eGFP (postsynaptic dendrite: eGFP only, 470/508–580; gate 1–6.0 ns, STED laser at 592 nm), and STAR440 (mouse-anti-gephyrin; 470/480–570; gate 1–6.0 ns, STED laser at 592 nm). STED laser intensity was adjusted according to the eGFP signal to enable clear visualization of dendritic structures and to reduce cross-activation during the detection of STAR440. Channel dye separation was performed to further split the eGFP and STAR440 signals. Images were recorded with LAS AF software (Leica Microsystems), analyzed using MetaMorph software (Molecular Devices), and figures were assembled in Adobe Photoshop CS6 and linear adjustments were done to correct black level and contrast.

##### LacZ reporter expression.

Using the LacZ reporter function of the mutated Cacna2d3 gene (Cacna2d3^tm1Dgen^; (Neely et al., 2010), β-galactosidase activity was analyzed in male α_2_δ-3^−/−^ knock-out mice. Eight-week-old mice were killed by CO_2_ asphyxiation, decapitated, and brains were removed from the skull. Subsequently, hemispheres were separated with a sagittal cut along the midline, placed medial side down on a flat piece of thin acryl glass, and frozen in 2-methylbutan (Carl Roth) cooled to −80°C. Native samples were stored at −80°C until further processing and transferred to −20°C 1 d before sectioning. Twenty-micrometer-thick sagittal sections of one hemisphere were obtained with a cryotome (NX50; Histocom), collected on polylysine-coated glass slides (Lactan), and stored at −20°C until further use. Sections were air dried at room temperature for 15 min, fixed with freshly prepared cold 4% pF and 0.2% glutaraldehyde (Sigma-Aldrich) for 10 min, and washed repeatedly in PBS for 30 min. X-Gal staining reaction was done with X-Gal (Boehringer Mannheim; 4% in N,N-dimethylformamide) diluted 1:40 in prewarmed X-Gal buffer (5 mm K_3_Fe(CN)_6_, 5 mm K_4_Fe(CN)_6_ · 3H_2_O, 2 mm MgCl_2_ in PBS; Sigma-Aldrich) at 37°C in a humidified incubator. Reaction was stopped after 24 h by several washes with PBS and rinsing in Milli-Q water (Millipore) at room temperature. Sections were counterstained with 0.2% eosin (Carl Roth) in 0.1 m CH_3_COOH in Milli-Q, dehydrated by a graded series of ethanol, followed by washing in xylene (Carl Roth) and mounting with Eukitt (Christine Gröpl, Tulln, Austria). Representative images were recorded with a BX53 microscope (Olympus) equipped with a SC100 color camera (Olympus). Eight-bit panorama photographs were created by scanning specimens with a 4× 0.16 NA objective and using the manual multiple image alignment (MIA) function in cellSens Dimension software (Olympus).

#### Image analysis and quantification

##### Colocalization of synaptic proteins.

The synaptic localization of HA-tagged α_2_δ subunits, presynaptic (synapsin, vGAT, vGLUT1) and postsynaptic proteins (GABA_A_R subtypes, gephyrin, GLUR1) were analyzed with the “line scan” function in MetaMorph software (Molecular Devices) (Di Biase et al., 2009). Average fluorescence intensities of green (A488), blue (A350), and red (A594) signals were measured along a line of 3 or 8 μm length, background subtracted, and plotted in Microsoft Excel.

##### Quantification of fluorescent clusters in single boutons.

To analyze the effects of presynaptic expression of α_2_δ subunits in GABAergic synapses of cultured MSNs, 14-bit grayscale images from triple-fluorescence labeling were acquired from the eGFP (green), GABA_A_R_β2/3_ (red), and vGAT (blue) channels. Images were analyzed with a custom programmed and semiautomated MetaMorph journal (Molecular Devices) as follows. Corresponding eGFP and vGAT images were superimposed, eGFP/vGAT-positive varicosities (putative GABAergic synapses) were randomly chosen as regions of interest (ROIs), and a region for background subtraction was selected. Axonal varicosities were defined as prominent swellings with higher fluorescence signal compared with the adjacent axonal shaft. Subsequently, eGFP, GABA_A_R, and vGAT grayscale images were thresholded to include the entire area of individual varicosities (eGFP) and synaptic clusters (GABA_A_R, vGAT). Selected ROIs were then transferred to the eGFP image and, by applying the “shrink region to fit” tool, automatic boundaries were drawn according to the threshold enabling only colocalized clusters to be analyzed. ROIs and background regions were further transferred to the thresholded GABA_A_R and vGAT channel images and ROI staining intensities were recorded. Therefore, the following parameters could be measured for the individual synapses in each of the channels in a blinded manner: eGFP threshold area as a measure for bouton size and average and integrated fluorescence intensities providing information on the size and intensity of clusters. To address the effects of presynaptic expression of α_2_δ subunits in glutamatergic synapses of α-Nrxn TKO neurons, 14-bit grayscale images from triple-fluorescence labeling were acquired from the eGFP (green), GABA_A_R_β2/3_ (red), and vGLUT1 (blue) channels. Images were analyzed with a custom programmed and semiautomated journal as described above. Corresponding eGFP and vGLUT1 images were superimposed and eGFP/vGLUT1-positive varicosities (putative glutamatergic synapses) were randomly selected as ROIs. To analyze the effects of presynaptic expression of α_2_δ subunits in glutamatergic synapses of cultured hippocampal WT neurons, 14-bit grayscale images from triple-fluorescence labeling were acquired from the eGFP (green), GLUR1 (red), and GABA_A_R_β2/3_/gephyrin (blue) channel. Images were analyzed in the same manner as mentioned above but without the use of a semiautomatic MetaMorph journal. For all experiments, background subtractions for individual channels and further data organization and analyses were done with Microsoft Excel. Mean values for individual cells were calculated from individual boutons and normalized to the control (eGFP only) or α_2_δ-2 condition within each culture preparation. Cumulative frequency distribution of GABA_A_R_β2/3_ fluorescence intensities of single boutons was done to visualize the effect of α_2_δ subunits on the whole GABA_A_R population. Two to four independent culture preparations were analyzed and details are given in the respective figure legends.

##### Quantification of gephyrin content in mismatched and neighboring synapses.

To compare gephyrin content of α_2_δ-2-induced mismatched synapses with endogenous clusters, 14-bit grayscale images from triple-fluorescence labelings were acquired from the eGFP (green), GLUR1 (red), and gephyrin (blue) channel and analyzed with MetaMorph software (Molecular Devices). First, corresponding eGFP, GLUR1, and gephyrin images were superimposed and eGFP-positive varicosities expressing α_2_δ-2 (putative mismatched synapses) were selected as ROIs and a region was selected for background subtraction. eGFP, GLUR1, and gephyrin grayscale images were thresholded to include the entire area of individual varicosities (eGFP) and synaptic clusters (GLUR1, gephyrin). Regions were transferred to the eGFP image, shrunk to fit the threshold area, and further transferred to the thresholded gephyrin channel images. After measuring the integrated gephyrin intensity of eGFP-positive mismatched synapses, ROIs of 10 untransfected neighboring WT synapses located on the same dendrite were selected and regions were shrunk according to the gephyrin threshold. The GLUR1 staining served as orientation to outline dendritic morphology of untransfected neurons. Per image, one mismatched gephyrin cluster induced by α_2_δ-2 was compared with the mean of 10 endogenous clusters. Dots in graph represent values for individual boutons (mismatched synapses) and means of 10 endogenous clusters measured per image. Values were normalized to endogenous gephyrin intensities of neighboring synapses within each culture preparation. Three independent culture preparations were done and eight to 10 images per preparation were analyzed.

##### Analysis of synapse localization in gSTED images.

Scans of mCherry-labeled boutons (control or α_2_δ-2) contacting eGFP-labeled postsynaptic dendrites were acquired from the eGFP (green, gSTED mode) and mCherry (red, confocal mode) channels. Axonal varicosities were visible as prominent swellings with higher fluorescence signal compared with the adjacent axonal shaft. Initially, linear contrast adjustments were done with LAS AF software (Leica Microsystems), thus enabling clear visualization of postsynaptic dendritic spines and the potential overlap of fluorescence signals, which was used to define the contact zone of putative synapses. Superimposed images were exported into a TIFF format and further analyzed using MetaMorph software (Molecular Devices). To determine the position of synapses on postsynaptic elements, two types of analysis were used. First, the postsynaptic contact sites of presynaptic boutons were manually classified as dendritic shafts, dendritic spines, or unclear localizations. Second, the distances from the postsynaptic dendritic shafts to the contact points with the presynaptic boutons were calculated as follows. First, the distances between the center of the dendritic shafts and the shaft surfaces were determined and measured. Second, the distances between the center of dendritic shafts and the contact zone (presynaptic bouton with postsynaptic dendrite) were determined and measured. Third, the actual distance from the dendritic shaft surface to the contact zone was determined by subtracting the individual center surface distances from the center contact zone distances. Dots in graph (Fig. 11*C*) represent values for individual boutons and means (lines) ± SEM are shown. Two independent culture preparations were analyzed, of which one experiment was performed in a blinded manner.

#### Electrophysiology

Induced (evoked) AMPAR-mediated synaptic responses were recorded from paired hippocampal neurons (holding potential −70mV) using the whole-cell patch-clamp technique. Neurons were stimulated with 10 ms depolarization pulse from a holding potential of −70 to 60 mV. Patch pipettes were pulled from borosilicate glass (Harvard Apparatus), fire-polished (Microforge MF-830; Narishige), and had resistances of 2.5–4 MΩ when filled with the following (in mm): 130 K-gluconate, 1 MgCl_2_, 10 HEPES, 5 EGTA, 4 Mg-ATP, and 0.3 Na-GTP, pH 7.2 with KOH. The bath solution contained the following (in mm): 137 NaCl, 3 KCl, 10 HEPES, 2 MgCl_2_, 1.8 CaCl_2_, 0.05 DL-AP5, and 10 glucose, pH 7.4 with NaOH. Currents were recorded with an EPC 10 amplifier controlled by PatchMaster software (HEKA Elektronik Dr. Schulze). To test synaptic plasticity (or paired-pulse plasticity), we applied a pair of pulses with varying interpulse intervals (10, 25, 50, 100, 250, or 500 ms) and calculated the paired-pulse amplitude ratio (P2/P1).

To test spontaneous activity of mixed cortical-MSN cultures, TTX (1 μm) and bicuculline (10 μm) or CNQX (10 μm) were added to bath solution and miniature EPSCs (mEPSCs; holding potential −70 mV) or miniature IPSCs (mIPSCs; holding potential 0 mV), respectively, were recorded.

#### Sequence alignment and homology modeling

Information on α_2_δ-2 splice sites was obtained from the Uniprot (Q6PHS9) and Ensemble (ENSMUSG00000010066) databases. Protein sequences of the distinct variants were aligned with CLUSTAL O (1.2.4) multiple sequence alignment tool. The high-resolution structure of α_2_δ-1 (PDB code: 5GJV; Wu et al., 2016) was used as a template to model the structure of α_2_δ-2 splice variants. The pdb files were generated with the Swiss-Model server (http://www.expasy.org/swissmod/SWISS-MODEL.html) and models were exported and further analyzed using UCSF Chimera (http://www.rbvi.ucsf.edu/chimera).

#### Experimental design and statistical analysis

According to the RRR principle, the number of mice used was kept to the minimum necessary for a statistical representative analysis, which was comparable to numbers reported in previous studies. Where indicated and possible, investigators were partially blinded during experiments and analyses. Moreover, analysis of presynaptic and postsynaptic proteins was done in a blinded manner as described above. Two to four independent culture preparations were analyzed per experiment and details on cell or bouton numbers are given in the respective figure legends. The graphs show values of individual cells/boutons (dots) and means (line) ± SEM. The numbers (*n*) used to calculate SEMs were either given by number of animals used (qRT-PCR) or the number of analyzed cells/boutons. Before statistical analysis, the distribution of datasets was evaluated with histograms. Whenever datasets were strongly skewed or normality was experimentally excluded (e.g., a large proportion of “0” values), raw data were log10 transformed and data distribution was reevaluated by histograms. For normally distributed raw or log10-transformed datasets, unpaired *t* test or one-way ANOVA with Holm–Sidak *post hoc* test was used. Alternatively, when log10 transformation did not result in normally distributed data, the Mann–Whitney *U* test or Kruskal–Wallis ANOVA with Dunn's *post hoc* analysis was applied. Significance levels (*p*-values) of statistical tests and *post hoc* analysis are presented in the respective figure legends. The model in Figure 3 was generated with Maya software (version 2018; Autodesk). Data, graphs and figures were organized, analyzed, and assembled using Microsoft Excel, GraphPad Prism 6, SigmaPlot (Systat Software), and Adobe Photoshop CS6.

## Results

### Presynaptic overexpression of α_2_δ-2 induces the formation of mismatched synapses in excitatory hippocampal neurons

**Figure 1.**
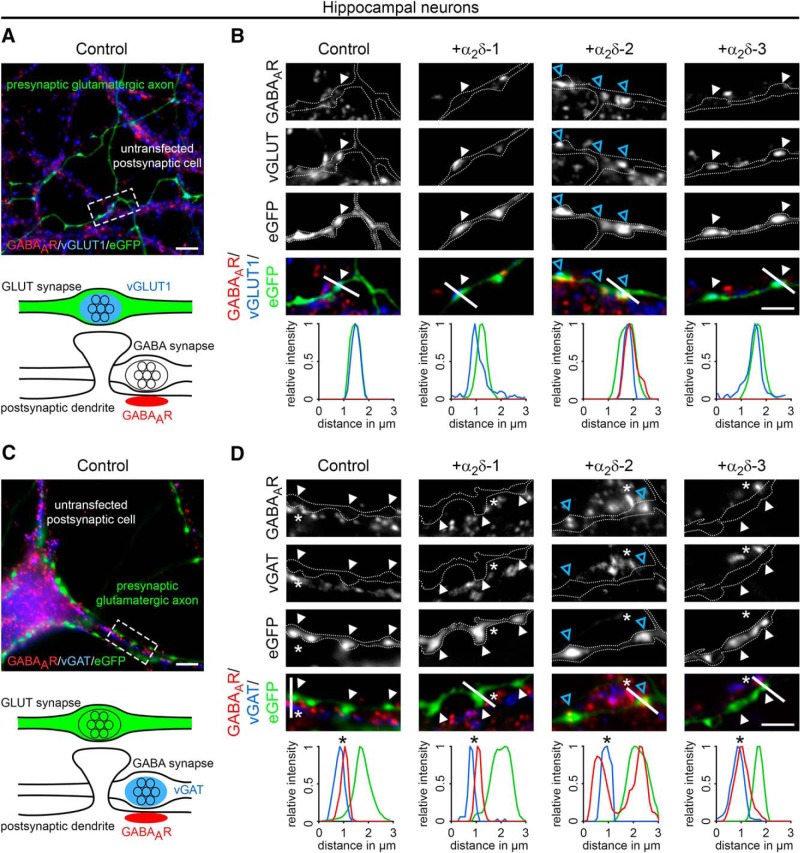
Presynaptic expression of α_2_δ-2 in cultured hippocampal neurons induces the formation of mismatched synapses. Representative immunofluorescence micrographs of cultured hippocampal neurons cotransfected with distinct α_2_δ subunits and soluble eGFP. Putative presynaptic en passent boutons (arrowheads) were identified as eGFP-filled axonal varicosities along dendrites of untransfected neurons (axons are outlined with dashed lines). ***A***, ***B***, Double labeling of transfected neurons (20–30 DIV) for vGLUT1 and the GABA_A_R _β2/3_ subunit. Colocalization of fluorescence signals was analyzed using line scans. ***A***, In control boutons (eGFP only), potential glutamatergic synapses are positive for presynaptic vGLUT1 but negative for postsynaptic GABA_A_R (summarized in sketch). ***B***, Presynaptic expression of α_2_δ-2 (blue arrowheads) induces postsynaptic GABA_A_R localization opposite transfected vGLUT1 positive nerve terminals. In contrast, postsynaptic GABA_A_Rs are not expressed opposite glutamatergic synapses expressing α_2_δ-1, α_2_δ-3, or eGFP only (control). ***C***, ***D***, Double labeling of transfected neurons (20–30 DIV) for vGAT and postsynaptic GABA_A_R. Colocalization of fluorescence signals was analyzed using line scans. ***C***, In control boutons (eGFP only), potential glutamatergic synapses are negative for presynaptic vGAT and postsynaptic GABA_A_R (summarized in sketch). ***D***, Transfected axonal varicosities were negative for vGAT in all conditions. Note the specific GABA_A_R labeling opposite vGAT-negative nerve terminals expressing α_2_δ-2 (blue arrowheads), which is in contrast to the colocalized fluorescence signals of vGAT and GABA_A_R in untransfected GABAergic neighboring synapses (asterisks). Representative images of two independent culture preparations are shown. Scale bars, 10 μm (***A***,***C***) and 3 μm (***B***,***D***).

To date, the synaptic functions of α_2_δ subunits have been identified in cells and tissues primarily expressing a single dominant α_2_δ isoform (Eroglu et al., 2009; Pirone et al., 2014; Fell et al., 2016; Wang et al., 2017). To study the involvement of the individual α_2_δ isoforms in glutamatergic synapse formation and differentiation, we homologously overexpressed mouse α_2_δ-1, α_2_δ-2, and α_2_δ-3 cDNAs in WT mouse hippocampal cultures together with soluble eGFP. To analyze the consequences on synapse differentiation, transfected neurons were immunolabeled against the vesicular glutamate transporter (vGLUT1), a marker for presynaptic excitatory synapses, and the postsynaptic GABA_A_R_β2/3_ subunit (GABA_A_R), which is typically absent in glutamatergic synapses. Immunofluorescence analysis revealed that, as expected, in control hippocampal neurons (eGFP only) axonal varicosities were positive for presynaptic vGLUT1 and negative for postsynaptic GABA_A_R labeling (Fig. 1*A*,*B*, first column). Surprisingly, presynaptic expression of α_2_δ-2 induced a robust localization of GABA_A_R clusters opposite vGLUT1-positive presynaptic boutons (Fig. 1*B*, blue arrowheads). These apparently mismatched synapses are atypical because they are formed between excitatory nerve terminals and inhibitory postsynaptic receptors. Importantly, mismatched postsynaptic receptor localization was not observed opposite terminals expressing α_2_δ-1 or α_2_δ-3.

Excitatory and inhibitory neurotransmitter transporters can coexist in glutamatergic hippocampal and cerebellar mossy fiber terminals, possibly leading to the corelease of glutamate and GABA and the localization of postsynaptic GABA_A_Rs in spines (Bergersen et al., 2003; Münster-Wandowski et al., 2016). Therefore, we next tested whether vesicular GABA transporters (vGATs) are present in synapses overexpressing presynaptic α_2_δ subunits. As expected and in contrast to neighboring untransfected GABAergic synapses (Fig. 1*D*, asterisks), presynaptic vGAT and postsynaptic GABA_A_Rs were not found in putative excitatory synapses expressing α_2_δ-1, α_2_δ-3, or eGFP only (Fig. 1*C*,*D*). Most importantly, presynaptic vGAT labeling was also absent in synapses expressing presynaptic α_2_δ-2, which again showed strong postsynaptic GABA_A_R clustering (Fig. 1*D*, +α_2_δ-2, blue arrowheads). Therefore, the mismatched localization of postsynaptic GABA_A_Rs cannot be explained by the existence of inhibitory neurotransmitters within glutamatergic nerve terminals.

**Figure 2.**
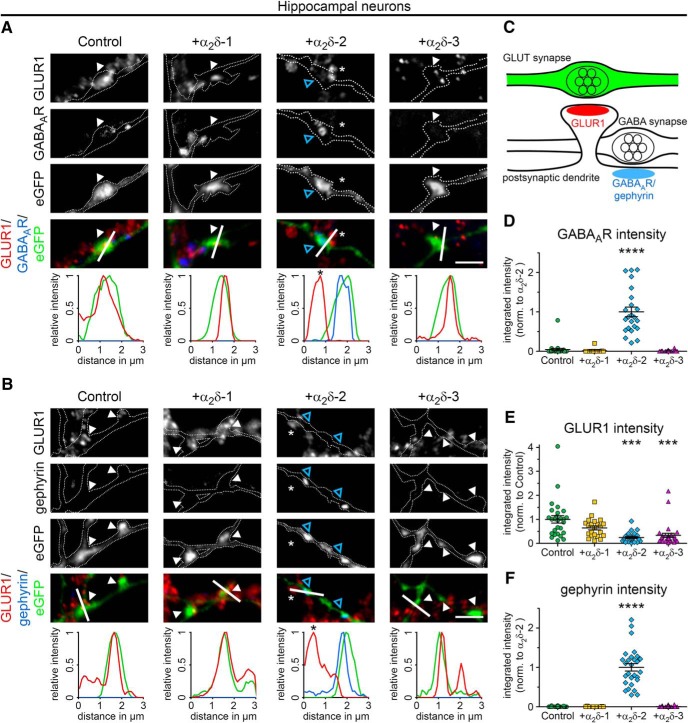
Presynaptic expression of α_2_δ-2 alters the postsynaptic composition of glutamatergic synapses. ***A***, ***B***, Representative immunofluoresence micrographs of cultured hippocampal neurons (24–30 DIV) cotransfected with distinct α_2_δ subunits and soluble eGFP (control condition with eGFP alone is summarized in sketch, **C**). Colocalization of fluorescence signals was analyzed using line scans. ***A***, Colabeling of the AMPA receptor subtype GLUR1 and the GABA_A_R. Presynaptic expression of α_2_δ-2 (blue arrowheads) induces postsynaptic GABA_A_R localization with a concomitant reduction of the GLUR1 fluorescence signal. In contrast, postsynaptic GABA_A_Rs are not expressed opposite putative glutamatergic synapses expressing α_2_δ-1, α_2_δ-3 or eGFP only (control). ***B***, Colabeling of GLUR1 and gephyrin, the scaffolding protein of GABAergic synapses. Presynaptic expression of α_2_δ-2 (blue arrowheads) induces postsynaptic gephyrin localization with a concomitant reduction of the GLUR1 fluorescence signal. In contrast, postsynaptic gephyrin immunoreactivity is absent opposite putative glutamatergic synapses expressing α_2_δ-1, α_2_δ-3, or eGFP only (control). ***D***–***F***, Quantitative analysis of GABA_A_R (***D***), GLUR1 (***E***), and gephyrin (***F***) fluorescence intensities. Values were normalized to α_2_δ-2 (***D***, ***F***) or eGFP only (control, ***E***) fluorescence intensities within each culture preparation. Note the significant increase of postsynaptic GABA_A_R (***D***) and gephyrin (***F***) clusters opposite presynaptic boutons expressing α_2_δ-2. In contrast, integrated intensity of GLUR1 was significantly reduced opposite axonal varicosities transfected with α_2_δ-2 or α_2_δ-3. Values for individual cells (dots) and means (line) ± SEM are shown. Data are shown from three independent culture preparations; 11–25 (***D***), 22–30 (***E***), and 11–28 (***F***) cells were analyzed in each condition. Statistics: ***D***, Kruskal–Wallis ANOVA with Dunn's *post hoc* analysis: *H*_(4)_ = 51.6, *p* < 0.0001, *post hoc*: ****p < 0.0001 between α_2_δ-2 and all other conditions; ***E***, ANOVA on log10-transformed data with Holm–Sidak *post hoc* analysis: *F*_(3,100)_ = 18.6, *p* < 0.001, *post hoc*: ***p < 0.001 between control/α_2_δ-1 and α_2_δ-2/α_2_δ-3; ***F***, Kruskal–Wallis ANOVA with Dunn's *post hoc* analysis: *H*_(4)_ = 64.6, *p* < 0.0001, *post hoc*: ****p < 0.0001 between α_2_δ-2 and all other conditions. Asterisks in graphs indicate the significant difference compared with control. Scale bars, 3 μm.

To further characterize our observation, we next quantitatively assessed the postsynaptic receptor composition of mismatched glutamatergic synapses by double labeling transfected hippocampal neurons with markers for excitatory and inhibitory synapses (Fig. 2*A–C*). Immunofluorescence analysis revealed that, in control hippocampal neurons (eGFP only) postsynaptic AMPARs (GLUR1), but not GABA_A_Rs, were expressed opposite transfected presynaptic terminals, as expected for putative glutamatergic synapses (Fig. 2*A*, control). Consistent with our initial observations (Fig. 1), postsynaptic GABA_A_R fluorescent intensities were strongly and significantly increased opposite α_2_δ-2-expressing presynaptic terminals (Fig. 2*A*,*D*). The elevated GABA_A_ receptor abundance (Fig. 2*A*) was accompanied by a 76% reduction of GLUR1 clustering compared with control (Fig. 2*E*). Surprisingly, presynaptic expression of α_2_δ-3, which did not alter postsynaptic GABA_A_R clustering, also caused a significant reduction of GLUR1 receptors (66% reduction vs control). Finally, we also observed a slight but not significant tendency for a reduced GLUR1 receptor expression opposite α_2_δ-1-overexpressing boutons (Fig. 2*E*). We next tested whether gephyrin, a key organizer of GABAergic synapses that anchors, clusters, and stabilizes GABA_A_R and other postsynaptic elements of inhibitory synapses (Choii and Ko, 2015), was also recruited to mismatched GABA_A_R-positive synapses. Indeed, we found that the strong increase in GABA_A_R expression opposite α_2_δ-2-expressing terminals was accompanied by similarly increased gephyrin abundance, whereas it was missing from control and α_2_δ-1- or α_2_δ-3-expressing synapses (Fig. 2*B*,*F*).

### Presynaptic α_2_δ-2 recruits synaptic GABA_A_R subtypes

**Figure 3.**
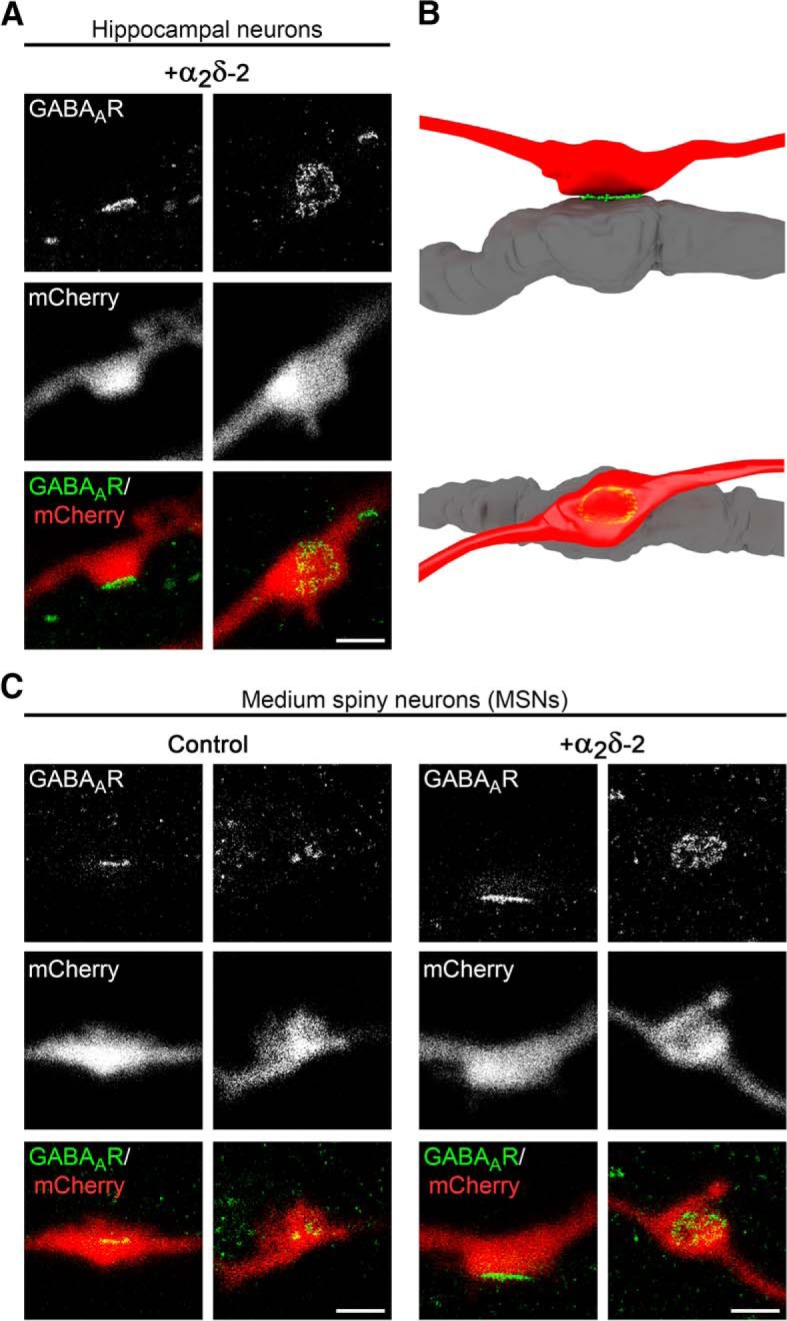
GABA_A_R clusters are confined to the postsynaptic membrane. gSTED micrographs of cultured hippocampal (***A***) or MSNs (***C***) transfected with α_2_δ-2 and mCherry or mCherry only (control; 20–30 DIV). Transfected neurons (red, detected in confocal mode) were immunolabeled for the GABA_A_R (green, detected in gSTED mode). In all conditions, postsynaptic GABA_A_R clusters are closely opposed to mCherry-positive presynaptic boutons. ***B***, 3D model showing that the GABA_A_R staining pattern depends on the orientation of the imaged synapse, which applies both for hippocampal as well as MSNs. Scale bar, 1 μm.

Because immunofluorescence analysis suggested an effect on postsynaptic GABA_A_R clusters opposite presynaptic boutons, we next used superresolution gSTED microscopy to verify their subsynaptic localization (Fig. 3*A*). Postsynaptic GABA_A_R clusters appear closely opposed to mCherry-positive, α_2_δ-2-overexpressing presynaptic boutons: depending on the orientation of the imaged synapses, the GABA_A_R-labeling pattern is either visible as a thin line (side view; Fig. 3*A*, first column and Fig. 3*B*, top) or as a ring-like structure (planar view; Fig. 3*A*, second column and Fig. 3*B*, bottom). These labeling patterns support a confined localization of α_2_δ-2-recruited GABA_A_Rs in the postsynaptic membrane.

**Figure 4.**
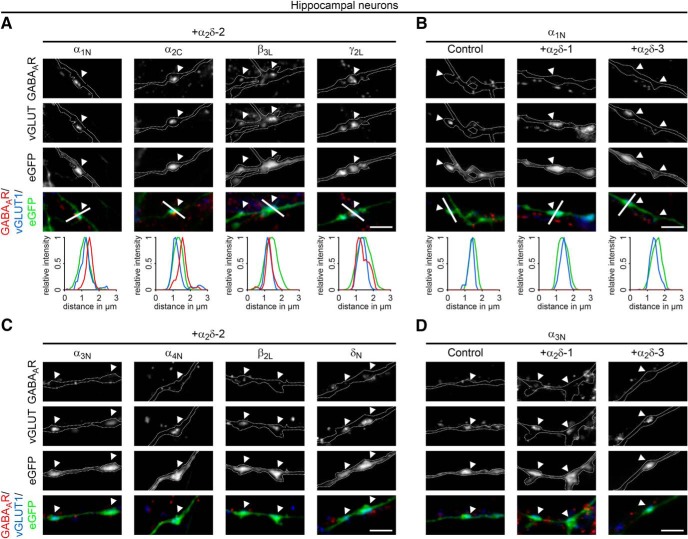
Presynaptic expression of α_2_δ-2 induces the recruitment of synaptic GABA_A_R subtypes. Representative immunofluorescence micrographs of cultured hippocampal neurons cotransfected with distinct α_2_δ subunits and soluble eGFP. Transfected permeabilized neurons (20–30 DIV) were double stained for vGLUT1 and different postsynaptic GABA_A_R subtypes. Colocalization of fluorescence signals within eGFP-filled axonal varicosities (arrowheads, axons are outlined with dashed lines) was analyzed using line scans. ***A***, ***B***, Immunofluorescence analysis identified intensely fluorescent clusters of the GABA_A_R subunits α_1_, α_2_, β_3_, and γ_2_ opposite α_2_δ-2 expressing glutamatergic (vGLUT1-positive) nerve terminals (***A***). In contrast, these postsynaptic GABA_A_R subtypes are absent opposite putative glutamatergic synapses expressing eGFP only (control), α_2_δ-1, or α_2_δ-3 (***B***, micrographs depict examples for α_1_). ***C***, ***D***, Labeling of the GABA_A_R subunits α_3_, α_4_, β_2_, and δ displayed weak and mainly extrasynaptic immunoreactivity in all conditions. Note that all GABA_A_R subtypes presented for α_2_δ-2 were also analyzed in hippocampal neurons expressing eGFP only, α_2_δ-1, and α_2_δ-3. Representative images of two independent cultures are shown. Scale bars, 3 μm.

GABA_A_Rs are pentameric ligand-gated chloride channels consisting typically of α, β, γ, and δ subunits (Hörtnagl et al., 2013). Because the antibody used in our colocalization experiments (Figs. 1, 2) detects only the GABA_A_R β2 and 3 subunits, we next analyzed whether and which additional GABA_A_R subtypes can be recruited by presynaptic α_2_δ-2. Immunofluorescence analysis revealed robust labeling of the typically synaptic GABA_A_R subunits α_1_, α_2_, β_3_, and γ_2_ opposite α_2_δ-2 transfected vGLUT1-positive nerve terminals (Fig. 4*A*). In contrast, the mainly extrasynaptic subunits α_3_, α_4_, β_2_, and δ (Hörtnagl et al., 2013) were either not detectable in these α_2_δ-2-expressing synapses or diffusely and extrasynaptically located (Fig. 4*C*). Importantly, mismatched postsynaptic receptor localization of all distinct subtypes was neither observed opposite terminals expressing eGFP only (control) nor those expressing α_2_δ-1 or α_2_δ-3 (Fig. 4*B*,*D*, examples for α_1_ and α_3_ subunits are shown). Because there was no apparent difference between postsynaptic α_1_, α_2_, β_3_, γ_2_, and β_2/3_ labeling, we continued using the commercially available and extensively characterized β_2/3_ antibody for all subsequent experiments (Goffin et al., 2010; Arama et al., 2015; Stefanits et al., 2018).

### Potential mechanisms explaining an α_2_δ-2-induced mismatched synapse formation

**Figure 5.**
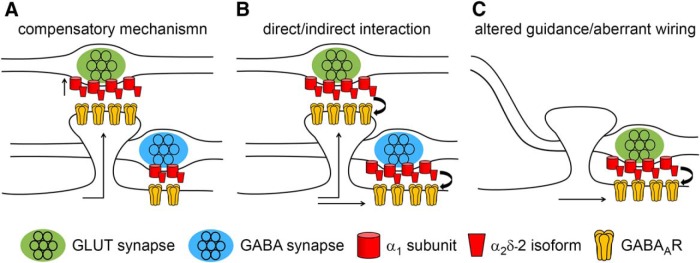
Potential mechanisms explaining the observed mismatched synapse formation of glutamatergic nerve terminals expressing α_2_δ-2. ***A***, Compensatory mechanism. Elevated expression of α_2_δ subunits increases presynaptic calcium channel abundance and current densities and thus glutamate release. Therefore, GABA_A_Rs could be recruited to the dendritic spine in an attempt to compensate for excessive excitatory synaptic activity. If this is the case, then GABA_A_R abundance at inhibitory synapses should not change upon α_2_δ-2 overexpression in GABAergic presynaptic terminals. ***B***, α_2_δ-2 may be involved in trans-synaptically anchoring postsynaptic GABA_A_Rs. In this scenario, GABA_A_R abundance should be increased when α_2_δ-2 is overexpressed in both glutamatergic and GABAergic synapses. ***C***, Trans-synaptic function of α_2_δ-2 could also induce aberrant axonal wiring by guiding glutamatergic axons to GABAergic postsynaptic locations positioned along dendritic shafts. This is in contrast to the normal situation in which glutamatergic synapses are generally formed on dendritic spines.

So far, our results show that the specific expression of a single α_2_δ subunit isoform, α_2_δ-2, in presynaptic glutamatergic terminals triggers a mismatched localization of postsynaptic GABA_A_Rs, which was accompanied by a strong reduction of postsynaptic AMPARs. The underlying mechanism could be explained by three hypotheses (Fig. 5): First, it is known that an elevated expression of α_2_δ subunits increases presynaptic calcium channel abundance and current densities (Hoppa et al., 2012; Geisler et al., 2015) and thus likely enhances synaptic transmission (Zhou and Luo, 2015; Chen et al., 2018). Therefore, postsynaptic GABA_A_Rs could be recruited opposite these glutamatergic boutons in an attempt to suppress excessive glutamatergic excitation (Shrivastava et al., 2011). If this hypothesis applies, then GABA_A_R abundance at inhibitory synapses should not change upon α_2_δ-2 overexpression in GABAergic presynaptic terminals (Fig. 5*A*). Second, presynaptic α_2_δ-2, an extracellular protein extending far into the synaptic cleft, may contribute to the anchoring of postsynaptic GABA_A_Rs by a trans-synaptic mechanism. Such a specific role of α_2_δ-2 should be independent of the synapse type and thus GABA_A_R abundance should be affected when α_2_δ-2 is overexpressed in both glutamatergic and GABAergic synapses (Fig. 5*B*). Independent of the two hypotheses, the observed reduction in GLUR1 labeling opposite α_2_δ-2-expressing glutamatergic terminals (Fig. 2) could be explained by GABA_A_Rs competing for AMPAR slots within dendritic spines. Alternatively, however, presynaptic α_2_δ-2 could also trans-synaptically induce an aberrant axonal wiring by guiding glutamatergic axons to GABAergic postsynaptic locations along dendritic shafts (Fig. 5*C*). In this scenario, the reduced GLUR1 expression would be a secondary effect because no GLUR1 receptors are to be expected in postsynaptic GABAergic synapses. To distinguish between these hypotheses, we next studied the consequence of presynaptic α_2_δ-2-overexpression on the molecular composition of GABAergic synapses.

### MSNs express three presynaptic α_2_δ isoforms

**Figure 6.**
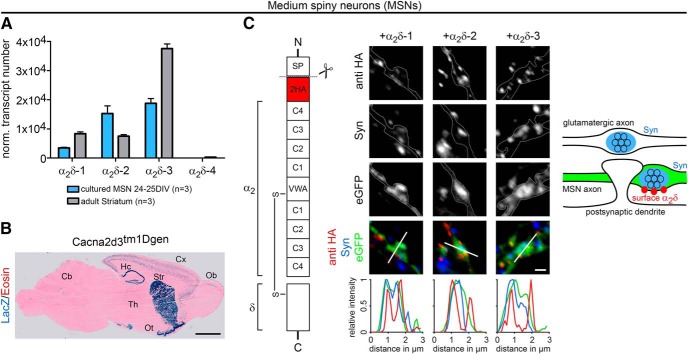
Striatum and cultured MSNs express three neuronal α_2_δ subunits. ***A***, Absolute qRT-PCR analysis revealed a stable expression of α_2_δ-1, α_2_δ-2, and α_2_δ-3 in adult mouse striatum and monocultured MSNs (24–25 DIV). Although α_2_δ-3 was the dominant isoform in striatal tissue, mRNA levels for α_2_δ-2 and α_2_δ-3 were similarly abundant in cultured MSNs. Error bars indicate mean ± SEM. Data from three independent culture/tissue preparations are shown. ***B***, β-galactosidase staining of sagittal cryosections of α_2_δ-3 knock-out mice carrying a LacZ cassette revealed intense labeling of striatum (Str), hippocampus (Hc), and olfactory tubercle (Ot). Lower expression was detected in the cortex (Cx), thalamus (Th), olfactory bulb (Ob), and parts of the cerebellum (Cb). ***C***, Schematic representation of the epitope-tagged α_2_δ subunits depicting the position of the extracellular 2HA tag inserted downstream of the signal peptide (SP), cache domains (C1–C4), and VWA (van Willebrand factor type A). Cultured MSNs were transfected with HA-tagged α_2_δ subunits together with soluble eGFP and live labeled with an antibody against the HA epitope at 24 DIV. All α_2_δ isoforms are expressed at the surface of presynaptic boutons, which is also shown by line scan analysis of α_2_δ-1, α_2_δ-2, and α_2_δ-3 (red) in relation to synapsin (blue) and eGFP (green). The sketch summarizes the observed labeling patterns. Representative images of three (***B***) and one (***C***) independent preparation(s) are shown. Statistics: ***A***, ANOVA on log10-transformed data with Holm–Sidak *post hoc* analysis: cultured MSNs: *F*_(3,8)_ = 460, *p* < 0.001; *post hoc*: *p* < 0.001 between all α_2_δ subunits except α_2_δ-2 vs α_2_δ-3 (*p* = 0.34); striatum: *F*_(3,8)_ = 891, *p* < 0.001; *post hoc*: *p* < 0.001 between all α_2_δ subunits except α_2_δ-1 vs α_2_δ-2 (*p* = 0.30). Scale bars, 2 mm (***B***) and 1 μm (***C***).

**Figure 7.**
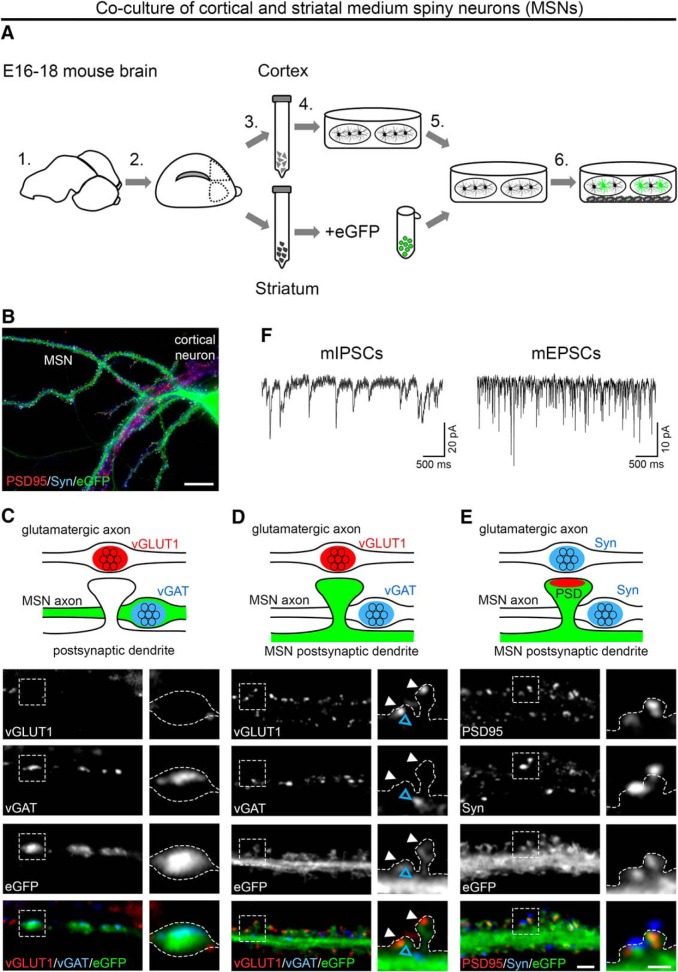
Presynaptic and postsynaptic differentiation of GABAergic MSNs. ***A***, Schematic illustration of the coculture procedure: Cerebral hemispheres were dissected and stripped of meninges (1). Parts of the prefrontal cortex and striatum were dissected as shown (2). Striatal and cortical tissue was collected separately and dissociated using trypsin-EDTA and trituration (3). Cortical neurons were plated on poly-l-lysine-coated coverslips while striatal neurons were transfected with soluble eGFP (4). Subsequently, striatal neurons were plated onto cortical neurons in a ratio of 3:2 (5) and maintained above a glial feeder layer (6). ***B***, Lentiviral infection or lipofection with soluble eGFP allowed discriminating MSNs from cortical neurons. ***C***–***E***, Double immunofluorescence of striatal–cortical cocultures (24–26 DIV) with presynaptic and postsynaptic markers for excitatory and inhibitory synapses. Neuronal morphology is outlined by eGFP. ***C***, GABAergic synapses of transfected eGFP-positive MSNs showed immunoreactivity for vGAT, whereas vGLUT1 was absent. ***D***, Axons of cortical neurons formed excitatory synapses on MSN spine heads (vGLUT1, white arrowheads), whereas GABAergic synapses were located on the dendritic shaft (vGAT, blue arrowhead). ***E***, Labeling of PSD-95 in spine heads opposite synapsin-positive presynaptic terminals further indicated the presence of functional excitatory synapses on MSNs. ***F***, Patch-clamp analysis of mIPSCs and mEPSCs in 14 DIV neurons confirmed the functionality of GABAergic and glutamatergic synapses. Representative micrographs of two independent cultures are shown. Scale bars, 50 μm (***B***), 3 μm (overview), and 1 μm (magnified selections; ***C***–***E***).

The cultures of hippocampal neurons used for the analysis of experiments presented in Figures 1, 2, 3, and 4 contain only 5–10% GABAergic interneurons (Obermair et al., 2003). Consequentially transfected GABAergic neurons are rarely observed. Therefore, to quantitatively address the role of α_2_δ subunits in GABAergic synapses, we established cultures of striatal neurons, which consist of ∼95% inhibitory MSNs. Quantitative RT-PCR revealed that differentiated monocultured MSNs (24–25 DIV) expressed three neuronal α_2_δ isoforms (α_2_δ-1, α_2_δ-2, and α_2_δ-3) similar to the adult mouse striatum (Fig. 6*A*). Whereas α_2_δ-2 and α_2_δ-3 expression levels were similar in cultured neurons, the higher abundance of α_2_δ-3 transcripts in 8-week-old striatal tissue was consistent with previous studies (Cole et al., 2005). The strong expression of α_2_δ-3 was confirmed on cryosections of a mouse line carrying a lacZ reporter gene in the Cacna2d3 locus using β-galactosidase histochemistry (Fig. 6*B*). The α_2_δ-4 isoform, which is predominantly present in the retina, was hardly detectable and thus not considered for the following experiments. For studying presynaptic α_2_δ subunit expression in differentiated GABAergic neurons, we adapted a coculture system of GABAergic MSNs with glutamatergic cortical neurons (Penrod et al., 2011) (Fig. 7*A*). Lentiviral infection of MSNs with soluble eGFP just before starting the coculture allowed discriminating them from excitatory cortical neurons (Fig. 7*B*) based on their eGFP fluorescence. Immunolabeling of presynaptic and postsynaptic markers for excitatory (vGLUT1, PSD-95) and inhibitory (vGAT) synapses demonstrated that glutamatergic and GABAergic synapses formed properly on dendritic spines and shafts, respectively (Fig. 7*C–E*). Moreover, patch-clamp recordings of spontaneous mEPSCs and mIPSCs in MSNs further confirmed the functionality of glutamatergic and GABAergic synapses in 14 DIV cocultures (Fig. 7*F*). Finally, live-cell labeling of MSNs cotransfected with HA-tagged α_2_δ subunits and soluble eGFP revealed that all α_2_δ isoforms are expressed on the surface of presynaptic axonal varicosities and thus can in theory contribute to synaptic functions in GABAergic neurons (Fig. 6*C*).

### Presynaptic overexpression of α_2_δ-2 upregulates postsynaptic GABA_A_Rs in inhibitory synapses

**Figure 8.**
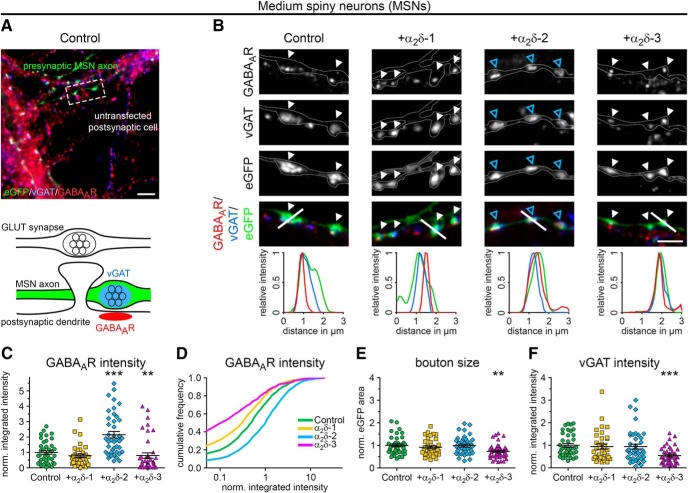
Presynaptic expression of α_2_δ-2 induces upregulation of postsynaptic GABA_A_Rs in MSNs. ***A***, ***B***, Representative immunofluorescence micrographs of cultured MSNs cotransfected with distinct α_2_δ subunits and soluble eGFP. Transfected neurons (21–28 DIV) were immunolabeled for vGAT and the GABA_A_R. Colocalization of fluorescence signals within eGFP-filled axonal varicosities (arrowheads, axons are outlined with dashed lines) was analyzed using line scans. ***A***, GABAergic synapses transfected with eGFP only (control) show matched presynaptic vGAT and postsynaptic GABA_A_R immunoreactivity (summarized in sketch). ***B***, Similar to control, postsynaptic GABA_A_Rs were localized opposite vGAT-positive presynaptic terminals expressing individual α_2_δ isoforms (see also colocalization in line scans). Most importantly, GABA_A_R clusters were larger and more intense opposite synaptic boutons expressing α_2_δ-2 (blue arrowheads). ***C***–***F***, Quantitative analysis of GABA_A_R fluorescence intensity (***C***), cumulative frequency distribution of GABA_A_R fluorescence intensity (***D***), bouton size (***E***), and vGAT fluorescence intensity (***F***). Values for individual cells (dots) and means (lines) ± SEM are shown. Values were normalized to the control within each culture preparation. Data from four independent culture preparations and 34–42 cells were analyzed in each condition. Statistics: ANOVA on log10-transformed data with Holm–Sidak *post hoc* analysis: ***C***, *F*_(3,152)_ = 17.6, *p* < 0.001; *post hoc*: ***p < 0.001 between α_2_δ-2 and control, ***p < 0.001 between α_2_δ-2 and α_2_δ-1/α_2_δ-3, **p = 0.004 between control and α_2_δ-3, *p* = 0.2 between control and α_2_δ-1; ***E***, *F*_(3,152)_ = 5.1, *p* < 0.01; *post hoc*: **p < 0.01 between α_2_δ-3 and control/α_2_δ-2, *p* = 0.08 between α_2_δ-3 and α_2_δ-1; ***F***, *F*_(3,152)_ = 8.0, *p* < 0.001; *post hoc*: ***p < 0.001 between α_2_δ-3 and control, **p < 0.01 between α_2_δ-3 and α_2_δ-1/α_2_δ-2. Asterisks in graphs indicate the significant difference compared with control. Scale bars, 10 μm (***A***) and 3 μm (***B***).

To distinguish between our hypotheses (Fig. 5), we coexpressed individual α_2_δ isoforms together with soluble eGFP in differentiated GABAergic MSNs. As expected, immunofluorescence analysis revealed that, in control MSNs (eGFP only), axonal varicosities were typically positive for presynaptic vGAT and postsynaptic GABA_A_R labeling (Fig. 8*A*,*B*). Postsynaptic GABA_A_Rs were similarly localized opposite vGAT-positive terminals expressing the individual α_2_δ isoforms (Fig. 8*B*). However, quantitative analysis of GABA_A_R fluorescence intensities of individual cells revealed that GABA_A_R labeling was strongly (∼2-fold) and significantly increased opposite boutons expressing the α_2_δ-2 isoform (Fig. 8*C*). Furthermore, presynaptic expression of α_2_δ-3 caused a 20% decrease of GABA_A_R labeling intensity compared with control (Fig. 8*C*). Plotting the cumulative frequency distribution of GABA_A_R fluorescence intensities of the single synapses demonstrates that presynaptic α_2_δ subunit isoforms differentially regulate postsynaptic GABA_A_R abundance in MSNs (Fig. 8*D*). GABA_A_R clusters were increased in size and intensity in the majority of synapses overexpressing α_2_δ-2 (blue line), causing a shift of the entire population to the right toward higher fluorescence intensities compared with control (eGFP only, green line). In contrast, ∼24% and ∼40% of all boutons expressing α_2_δ-1 (yellow line) and α_2_δ-3 (magenta line), respectively, lacked postsynaptic GABA_A_R labeling and the remaining population was left shifted toward smaller clusters. The slightly but significantly reduced GABA_A_R clustering opposite α_2_δ-3-overexpressing boutons (Fig. 8*C*) was associated with smaller presynaptic boutons (27% smaller than control; Fig. 8*E*) and decreased presynaptic vGAT content (46% lower than control; Fig. 8*F*). Conversely, presynaptic expression of α_2_δ-2, which strongly increased postsynaptic GABA_A_R clustering, affected neither bouton size nor vGAT labeling intensity.

Similar to mismatched glutamatergic synapses (see above), we used superresolution gSTED microscopy to visualize the subsynaptic localization of postsynaptic GABA_A_R clusters in cultured MSNs (Fig. 3*C*). MSNs were cotransfected with α_2_δ-2 and soluble mCherry or mCherry only (control). In both conditions, postsynaptic GABA_A_R clusters appeared closely opposed to mCherry-positive presynaptic boutons, thus confirming their localization in the postsynaptic membrane. Moreover, similar to hippocampal neurons, the GABA_A_R pattern was either visible as a thin line (side view; Fig. 3*C*, first and third column and Fig. 3*B*, top) or a ring-like structure (planar view; Fig. 3*C*, second and fourth column and Fig. 3*B*, bottom). Importantly, gSTED imaging further confirmed that presynaptic expression of α_2_δ-2 increased GABA_A_R density at the corresponding postsynaptic side.

### α-Nrxns modulate postsynaptic GABA_A_R abundance set by presynaptic α_2_δ-2

**Figure 9.**
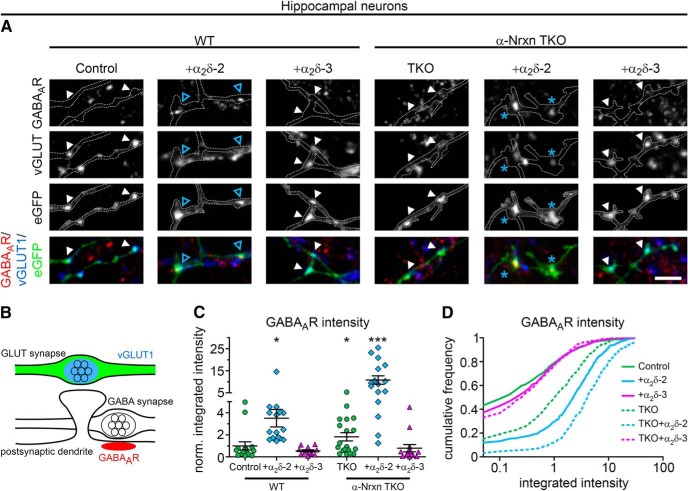
α-Nrxn modulates the effect of α_2_δ-2 on GABA_A_Rs. Representative immunofluorescence micrographs of cultured hippocampal neurons (20–25 DIV) prepared from WT or α-Nrxn TKO mice. ***A***, ***B***, In control boutons (eGFP only), potential glutamatergic synapses are positive for presynaptic vGLUT1 but negative for postsynaptic GABA_A_R staining (summarized in sketch, ***B***). Consistent with our initial observation (see Fig. 1), presynaptic expression of α_2_δ-2 (blue arrowheads) induces postsynaptic GABA_A_R localization opposite vGLUT1-positive nerve terminals in WT neurons. Most importantly, in α-Nrxn TKO synapses, the expression of α_2_δ-2 strongly induced postsynaptic GABA_A_R localization (blue asterisks). ***C***, Postsynaptic GABA_A_Rs clusters (integrated intensities) are significantly increased opposite glutamatergic nerve terminals expressing α_2_δ-2 compared with control (eGFP only). This effect is much stronger in α-Nrxn TKO synapses compared with WT neurons (3-fold higher GABA_A_R fluorescence intensity). ***D***, Cumulative frequency distribution further reveals that the vast majority of glutamatergic boutons are positive for postsynaptic GABA_A_Rs in WT+α_2_δ-2 (88%) and TKO+α_2_δ-2 (97%) and that the population is shifted toward larger and more intense clusters. However, this analysis also demonstrates that mismatched GABA_A_Rs already form opposite α-Nrxn TKO synapses, although at a lower overall intensity. Values for individual cells (dots) and means (lines) ± SEM are shown. Values were normalized to the WT control within each culture preparation. Data from two independent culture preparations and 12–19 cells were analyzed within each condition. Statistics: ***C***, ANOVA on log10-transformed data with Holm–Sidak *post hoc* analysis: genotype: *F*_(1,86)_ = 5.3, *p* = 0.023; transfection: *F*_(2,86)_ = 49.0, *p* < 0.001; genotype × transfection: *F*_(2,86)_ = 3.7, *p* = 0.03; *post hoc*: *p < 0.05, ***p < 0.001. Asterisks in graphs indicate the significant difference compared with control. Scale bar, 3 μm.

Thus far, our results revealed that presynaptic α_2_δ-2 strongly induced postsynaptic GABA_A_ receptor clustering in both glutamatergic and GABAergic synapses, suggesting that α_2_δ-2 acts trans-synaptically according to our hypothesis outlined in Figure 5. This means that α_2_δ-2 could either directly interact with postsynaptic receptors or may act via other synaptic organizer molecules such as α-Nrxns. Presynaptic α-Nrxns are synaptic cell adhesion molecules whose general importance in a variety of synaptic functions is well established (Reissner et al., 2013; Südhof, 2017). Several lines of evidence implicate α-Nrxns as interesting candidates for mediating a possible indirect interaction between α_2_δ-2 and GABA_A_Rs. First, α-Nrxns have been linked functionally to high-voltage-dependent Ca^2+^channels, which has strong effects on presynaptic neurotransmitter release (Missler et al., 2003; Chen et al., 2017). Second, α-Nrxns have been implicated as organizing molecules at both excitatory glutamatergic and inhibitory GABAergic synapses by promoting clustering of postsynaptic AMPARs (Heine et al., 2008; Aoto et al., 2013, 2015) and GABA_A_Rs (Kang et al., 2008). Third, the presynaptic complex of α-Nrxn and its ligand neurexophilin-1 is characteristic for inhibitory interneurons and can induce postsynaptic GABA_A_R recruitment when ectopically expressed at excitatory synapses (Born et al., 2014). Fourth, neurexin-1α has recently been shown to modulate P/Q-type (Brockhaus et al., 2018) and N-type (Tong et al., 2017) calcium channels by cooperating with α_2_δ subunits. Therefore, to test the possibility that upregulation of postsynaptic GABA_A_Rs depends on α-Nrxns, we expressed α_2_δ-2 in glutamatergic synapses lacking all three α-Nrxn isoforms (Missler et al., 2003). We transfected eGFP alone or in combination with α_2_δ-2 or α_2_δ-3 in hippocampal cultures from WT and α-Nrxn TKO mice. Immunofluorescence analysis revealed that, as expected, in control hippocampal neurons, axonal varicosities were typically positive for presynaptic vGLUT1 but negative for postsynaptic GABA_A_R labeling (Fig. 9*A*,*C*, WT control). Consistent with our previous observations, presynaptic expression of α_2_δ-2 induced the formation of mismatched synapses (Fig. 9*A*,*C*, WT+α_2_δ-2), whereas expression of α_2_δ-3 had no effect (*p* = 0.64; Fig. 9*A*,*C*, WT+α_2_δ-3). Strikingly, expression of presynaptic α_2_δ-2 in α-Nrxn TKO neurons induced postsynaptic GABA_A_R clustering opposite glutamatergic boutons more strongly than in WT (Fig. 9*A*,*C*, α-Nrxn TKO+α_2_δ-2). Quantitative analysis revealed that this effect was three times higher compared with the WT+α_2_δ-2 condition, suggesting that the propensity of α_2_δ-2 in inducing mismatched synapses was enhanced in the absence of α-Nrxns. In fact, increased GABA_A_R abundance was already visible in nontransfected α-Nrxn TKO synapses in which the presence of endogenous α_2_δ-2 may be responsible for a significantly increased baseline level of mismatched GABA_A_Rs (*p* = 0.023; Fig. 9*A*,*C*). This is an important result because it suggests that no overexpression of α_2_δ-2 is needed to induce postsynaptic GABA_A_R clustering, but an imbalance between α_2_δ-2 and α-Nrxns. These differential effects can be best compared by blotting the cumulative frequency distribution of GABA_A_R fluorescence intensities of all individual synapses analyzed (Fig. 9*D*). Compared with WT (control, green line), the entire population of synapses was right-shifted toward larger GABA_A_R clusters in α-Nrxn TKO neurons (dashed green line). Presynaptic expression of transfected α_2_δ-2 strongly shifted the distributions toward larger GABA_A_R clusters in WT (+α_2_δ-2, blue line) and α-Nrxn TKO neurons (TKO+α_2_δ-2, dashed blue line). Presynaptic expression of α_2_δ-3 had no effect in WT neurons, which, due to the lack of mismatched synapses, basically displayed only background GABA_A_R intensity (+α_2_δ-3, magenta line). Interestingly, overexpression of α_2_δ-3 in α-Nrxn TKO neurons reduced the elevated GABA_A_R clusters to control levels (TKO+α_2_δ-3, dashed magenta line). This possibly indicates that overexpressed, abundant α_2_δ-3 blocked the effect by endogenous α_2_δ-2 on baseline GABA_A_R intensity in TKO synapses. Together, our data suggest that presynaptic α_2_δ-2 potently induced mismatched synapse formation with GABA_A_R clustering in the absence of α-Nrxns. This demonstrates that the trans-synaptic role of α_2_δ-2 in recruiting postsynaptic GABA_A_Rs is independent of α-Nrxns. However, the fact that GABA_A_R abundance was already increased in α-Nrxn TKO synapses without overexpression of α_2_δ-2 and that the abundance could be differentially modulated by presynaptic α_2_δ-2 and α_2_δ-3 suggests a cooperative and trans-synaptic activity of α_2_δ subunits and α-Nrxns in fine-tuning postsynaptic GABA_A_R levels.

### α_2_δ-2 induces the aberrant wiring of glutamatergic axons to GABAergic postsynaptic positions

Our experiments described above demonstrate that presynaptic α_2_δ-2 induces the recruitment of postsynaptic GABA_A_Rs opposite glutamatergic and GABAergic presynaptic boutons. Moreover, this effect of α_2_δ-2 is independent of the presynaptic neurotransmitter identity, suggesting a trans-synaptic activity in recruiting/anchoring postsynaptic GABA_A_Rs. As hypothesized (Fig. 5), such a trans-synaptic role of α_2_δ-2 could result in two types of mismatched synapses: the GABA_A_Rs could either be recruited to postsynaptic compartments of dendritic spine synapses with a concomitant reduction of the resident glutamate receptors or α_2_δ-2 could induce an altered wiring of glutamatergic axons to GABAergic postsynaptic locations along the dendritic shaft.

**Figure 10.**
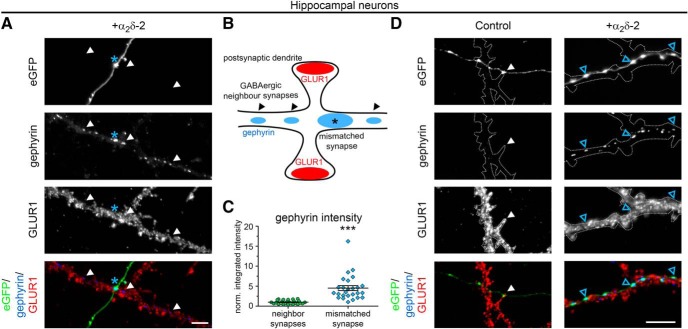
Mismatched synapses show characteristics of GABAergic shaft synapses. ***A***, ***D***, Representative immunofluorescence micrographs of cultured hippocampal neurons cotransfected with α_2_δ-2 and soluble eGFP. Colabeling of transfected neurons (24 DIV) for postsynaptic GLUR1 and gephyrin is shown. ***A***, Presynaptic expression of α_2_δ-2 induces a strong upregulation of postsynaptic gephyrin (asterisk) compared with neighboring endogenous GABAergic synapses situated on the same dendrite (arrowheads). Although GLUR1 expression was reduced in mismatched synapses (*; see Fig. 2), it was absent in endogenous GABAergic synapses (arrowheads, see sketch in ***B***). ***C***, Quantitative analysis of gephyrin fluorescence intensities in GABAergic neighboring and mismatched synapses. Dots represent values for individual boutons (mismatched synapses) and means of 10 endogenous clusters measured per image. Results were normalized to endogenous gephyrin intensities of neighboring synapses within each culture preparation. Data from three independent culture preparations are shown. Statistics: *t* test on log10-transformed data: *t*_(50)_ = 9.5; ***p < 0.001. ***D***, Left, Control boutons transfected with eGFP only form glutamatergic synapses on dendritic spines (gephyrin negative, GLUR1 positive; white arrowheads). Right, Expression of α_2_δ-2 in putative glutamatergic boutons induces the formation of mismatched synapses (gephyrin-positive, GLUR1-negative) along the shaft of the dendrite (blue arrowheads). Dendritic morphology of untransfected neurons was outlined according to GLUR1 labeling. Scale bars, 5 μm (***A***) and 4 μm (***B***).

**Figure 11.**
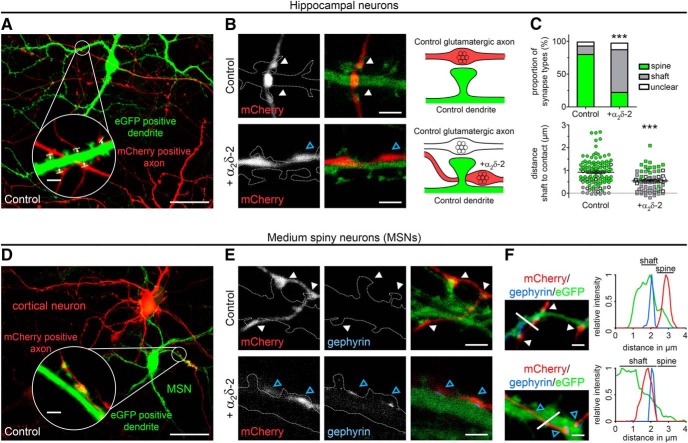
Presynaptic α_2_δ-2 induces the aberrant wiring of glutamatergic axons to dendritic shafts. ***A***, ***D***, To analyze the position of α_2_δ-2-induced mismatched synapses on postsynaptic dendrites of excitatory (***A***, hippocampal neurons) and inhibitory neurons (***D***, MSNs), presynaptic (control or α_2_δ-2) and postsynaptic neurons were labeled with mCherry (red) and eGFP (green), respectively (for details, see Materials and Methods). The magnified insets in ***A*** and ***D*** demonstrate the close association of presynaptic axonal varicosities (mCherry) with postsynaptic dendritic spines (eGFP), as expected for excitatory spine synapses. Using gSTED microscopy, the distance from the dendritic shaft to the contact zone of each synaptic bouton was measured (***A***, arrows and lines in the magnified inset). ***B***, ***E***, gSTED microscopy confirms the preferential location of excitatory synapses on dendritic spines of both glutamatergic or GABAergic neurons (white arrowheads and sketch in ***B***) and, most importantly, suggests an aberrant wiring of putative glutamatergic axons expressing α_2_δ-2 with postsynaptic sites along dendritic shafts (blue arrowheads and sketch in ***B***), as typically observed for GABAergic synapses. ***C***, Corresponding postsynaptic location of each contacting bouton was categorized as spine (green), shaft (gray), or as unclear (white; top) and the distance from the dendritic shaft to the contact point was measured (bottom). Although the vast majority of synaptic contact points in the control condition are located on dendritic spines (80%), presynaptic expression of α_2_δ-2 shifted the preferential contact points to dendritic shafts (65%, top). This is further confirmed by a significantly decreased contact point to shaft distance of α_2_δ-2-expressing compared with control synapses. Values for individual boutons (dots) and means (lines) ± SEM are shown. Dot colors (bottom) show the respective categorized synapse position (spine, green; shaft, gray; and unclear, white) presented in the top. Data from two independent culture preparations and 118 (control) and 75 (+α_2_δ-2) boutons were analyzed. Statistics: ***C***, top: χ^2^ test: χ^2^_(2)_ = 63.4, ***p < 0.001; bottom: Mann–Whitney *U* test: ***p < 0.001. ***E***, ***F***, gSTED microscopy (***E***) and high-resolution fluorescence microscopy (***F***) revealed that, similar to hippocampal neurons, presynaptic expression of α_2_δ-2 in cortical neurons shifted the preferential contact points to dendritic shafts of GABAergic MSNs, as indicated by gephyrin labeling and the dendritic position (blue arrowheads and line scan in ***F***). Representative micrographs of two independent cultures are shown. Scale bars, 50 μm (overview), 3 μm (insets, ***A***, ***D***), and 2 μm (***B***, ***E***, ***F***).

To distinguish between these two possibilities, we focused more closely on the position of the mismatched synapses along the postsynaptic neurons. First, it is important to emphasize that α_2_δ-2 induced the upregulation of postsynaptic GABAergic components only at the sites contacted by the presynaptic axon without affecting the neighboring synapses. This is best visualized by the observation that gephyrin fluorescence intensity was 4.5 times higher in α_2_δ-2-induced mismatched synapses (Fig. 10*A–C*, asterisks in *A* and *B*) compared with neighboring endogenous GABAergic synapses situated on the same dendrite, which are formed by axons from untransfected neurons (Fig. 10*A–C*, arrowheads in *A* and *B*). This finding further supports a local and synapse-specific effect of presynaptic α_2_δ-2 rather than a global alteration in the expression of GABAergic postsynaptic components. Importantly, α_2_δ-2-overexpressing glutamatergic axons preferentially formed synapses at typical GABAergic postsynaptic locations, namely linearly aligned along the dendritic shaft (Fig. 10*D*, right column, blue arrowheads), in contrast to control axons, which preferably contacted dendritic spines (Fig. 10*D*, left column, white arrowheads). Therefore, to determine the exact position of mismatched synapses on postsynaptic dendrites, we established hippocampal cultures in which presynaptic (control or α_2_δ-2) and postsynaptic neurons were transfected with mCherry (red) and eGFP (green), respectively (Fig. 11*A–C*). gSTED microscopy allowed precise visualization of the contact point between the presynaptic mCherry-positive axon and the postsynaptic eGFP-positive dendrite (Fig. 11*A*,*B*). As expected for glutamatergic excitatory synapses, the vast majority of axonal varicosities was located on dendritic spines in the control condition (80% spine, 13% shaft, 7% unclear; Fig. 11*B*,*C*, top). In contrast, presynaptic expression of α_2_δ-2 significantly shifted the preferential contact points to dendritic shafts (24% spine, 65% shaft, 11% unclear; χ^2^ test: *p* < 0.001). This finding was further corroborated by a highly significant reduction of the mean distance from the contacting presynaptic boutons to the postsynaptic dendritic shafts in α_2_δ-2-transfected compared with control axons (mean distance ± SEM: control: 0.92 ± 0.05 μm; α_2_δ-2: 0.54 ± 0.05 μm; Mann–Whitney *U* test: *p* < 0.001; Fig. 11*C*, bottom). Importantly, applying the same methodological approach for the coculture of GABAergic MSNs and glutamatergic cortical neurons (Fig. 11*D*, also see Fig. 7) revealed that axonal varicosities from control cortical neurons generally contacted dendritic spines of postsynaptic inhibitory MSNs (total of 65 synapses: 83% spine, 1.5% shaft, 15.5% unclear: Fig. 11*E*,*F*, top). In contrast, the presynaptic expression of α_2_δ-2 shifted the preferential contact points of cortical boutons to dendritic shafts of MSNs (total of 51 synapses: 19.6% spine, 72.6% shaft, 7.8% unclear; Fig. 11*E*,*F*, bottom, gephyrin labeling and position at dendritic shaft). These findings demonstrate that the α_2_δ-2-induced aberrant wiring is independent of the identity of the postsynaptic neuron.

Together, our results provide strong evidence that overexpression of presynaptic α_2_δ-2 induces the aberrant wiring of glutamatergic axons to postsynaptic GABAergic locations, resulting in a mismatch of presynaptic excitatory neurotransmitters and postsynaptic inhibitory receptors.

### Reduced synaptic transmission in aberrantly wired synapses

**Figure 12.**
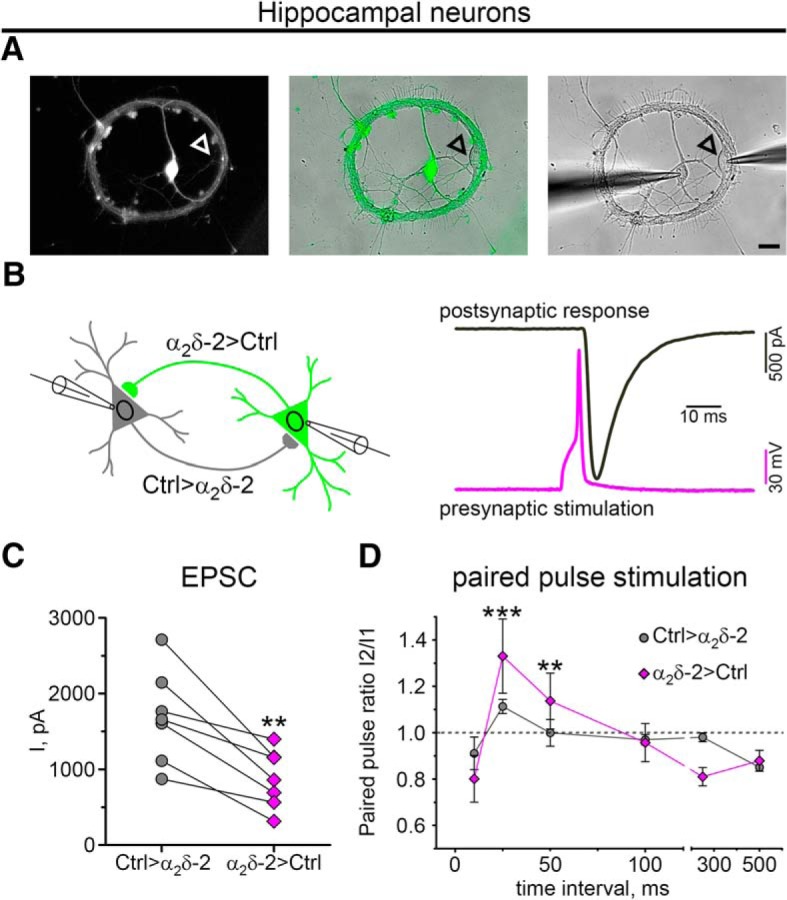
Presynaptic expression of α_2_δ-2 affects synaptic transmission in aberrantly wired synapses. ***A***–***C***, Experimental paradigm to study the functional consequences of presynaptic α_2_δ-2 overexpression in mismatched glutamatergic synapses of cultured hippocampal neurons. ***A***, Neurons were cultured in pairs of two synaptically connected cells in which one represents an untransfected control neuron (arrowhead) and the other an α_2_δ-2-overexpressing neuron (eGFP labeled). ***B***, Whole-cell patch-clamp recordings of excitatory synaptic transmission (13–18 DIV). An action potential in neuron 1 elicited by a given AP waveform recorded from WT hippocampal neurons caused eEPSCs in neuron 2. Synaptic transmission was analyzed in both directions of synaptically connected pairs. Therefore, putative glutamatergic spine synapses (control innervating α_2_δ-2-overexpressing neuron) and putative mismatched synapses (α_2_δ-2 overexpressing innervating control neuron) could be directly compared within the same pair. ***C***, ***D***, Quantitative analysis of mean eEPSC peak amplitudes (***C***) and paired-pulse response ratios (PPRs) as a measure for synaptic plasticity (***D***). ***C***, eEPSCs were strongly reduced (48%) in mismatched synapses. ***D***, Dashed line shows the boundary between paired-pulse depression (PPR < 1) and facilitation (PPR > 1). Note the slight significant increase in facilitation in mismatched synapses at 25 and 50 ms compared with the initial 10 ms interval. Values for individual pairs (dots) and means (lines) ± SEM are shown. Data from three independent culture preparations from seven (***C***) and six (***D***) pairs were analyzed. Statistics: ***C***, paired *t* test: *t*_(6)_ = 4.6; **p < 0.01; ***D***, two-way repeated-measures ANOVA: condition: *F*_(1,45)_ = 0.02, *p* = 0.9; interval: *F*_(5,45)_ = 11.1, *p* < 0.001; condition × interval: *F*_(5,45)_ = 2.7, *p* = 0.03; Holm–Sidak *post hoc* analysis: **p = 0.004, ***p < 0.001 compared with 10 ms within α_2_δ-2 to control. Scale bar, 20 μm.

Excitatory and inhibitory synapses require matched presynaptic and postsynaptic specializations to enable efficient and synchronous neurotransmission. To assess the functional consequences of α_2_δ-2 overexpression in presynaptic boutons of aberrantly wired glutamatergic synapses, we performed whole-cell patch-clamp recordings on synaptically connected pairs of isolated neurons grown on poly-l-lysine dots (Fig. 12*A*). We first investigated whether AMPAR-mediated evoked EPSCs (eEPSCs) between individual pairs of hippocampal neurons were altered by the lentiviral overexpression of α_2_δ-2 in the presynaptic neuron. Isolated synaptic currents were measured in the postsynaptic neuron in response to an action potential induced in the presynaptic neuron. The presynaptic action potential followed a given waveform recorded from WT hippocampal neurons (Fig. 12*B*). This experimental setup allowed to directly compare synaptic transmission in glutamatergic control synapses (presynaptic cell = control, postsynaptic cell = α_2_δ-2 overexpressing) and mismatched synapses (presynaptic cell = α_2_δ-2 overexpressing, postsynaptic cell = control). In each of seven neuronal pairs recorded, the eEPSC amplitude was lower in the mismatched compared with the control pair (on average reduced by 48%) (Fig. 12*C*). To discriminate whether the eEPSC reduction is the result of the reduced postsynaptic AMPA receptor expression associated with the aberrantly wired axons or if presynaptic α_2_δ-2 reduces release probability, we next measured synaptic paired-pulse response ratios (PPRs) (Katz and Miledi, 1968; Zucker and Regehr, 2002). eEPSCs were recorded in the postsynaptic neuron in response to two action potentials elicited in the presynaptic neuron at different interstimulus intervals. As previously reported for hippocampal pyramidal neurons, calculated PPRs displayed facilitation (Fig. 12*D*) with a decline in the magnitude at increasing intervals (Dobrunz et al., 1997). Overexpression of α_2_δ-2 in presynaptic neurons slightly increased facilitation at 25 and 50 ms compared with the initial 10 ms interval. The strong reduction of eEPSC amplitudes is thus consistent with a reduced number of functional synapses caused by the aberrant wiring of glutamatergic α_2_δ-2-expressing boutons with GABAergic postsynaptic sites. Conversely, the only moderately altered paired-pulse facilitation (PPF) indicates that the decreased amplitude of eEPSCs was not caused by a reduced presynaptic release probability. The observed slightly increased PPF may reflect an increased abundance of presynaptic calcium channels caused by the overexpression of α_2_δ subunits, as has been previously demonstrated (Hoppa et al., 2012).

### Lack of exon 23 in α_2_δ-2 splice variants mediates the trans-synaptic effect on GABA_A_Rs

**Figure 13.**
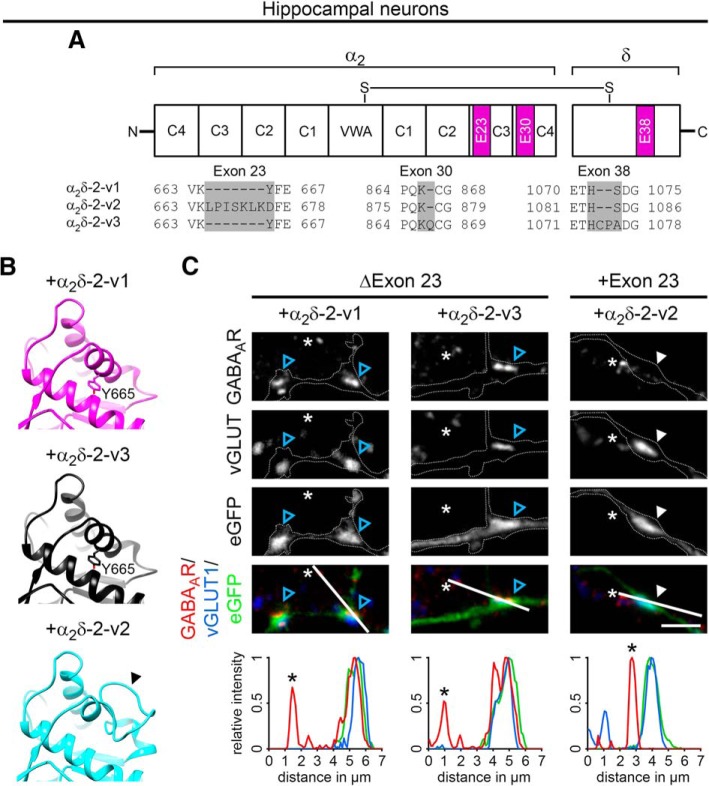
Lack of exon 23 in α_2_δ-2 splice variants mediates the trans-synaptic effect on GABA_A_Rs. ***A***, Schematic overview illustrating the approximate positions of three alternatively spliced regions of α_2_δ-2. Sequence alignment between α_2_δ-2-v1, the original construct used in this study, and two additional α_2_δ-2 variants (v2, v3) reveals alternative splicing of exons 23, 30, and 38. ***B***, Using homology modeling, we tested the potential consequences of alternative splicing on the structure prediction based on the high-resolution structure of α_2_δ-1 (PDB code: 5GJV; Wu et al., 2016; see Fig. 14). Inclusion of exon 23 in α_2_δ-2-v2 suggested the formation of an extra loop (arrowhead) leading to the disruption of an α-helix present in α_2_δ-2-v1 and α_2_δ-2-v3. ***C***, Representative immunofluorescence micrographs of cultured hippocampal neurons (21–26 DIV) cotransfected with distinct α_2_δ-2 splice variants and soluble eGFP. Presynaptic expression of either α_2_δ-2-v1 (original construct) or α_2_δ-2-v3, which both lack exon 23, robustly induces a mismatched GABA_A_R localization opposite vGLUT1-positive nerve terminals (blue arrowheads). In contrast, presynaptic expression α_2_δ-2-v2, which contains exon 23, failed to induce mismatched synapse formation (white arrowheads, line scans). Asterisks mark endogenous GABA_A_R clusters from untransfected neighboring synapses not colocalizing with presynaptic vGLUT1. Representative images of two independent cultures are shown. Scale bar, 3 μm.

**Figure 14.**
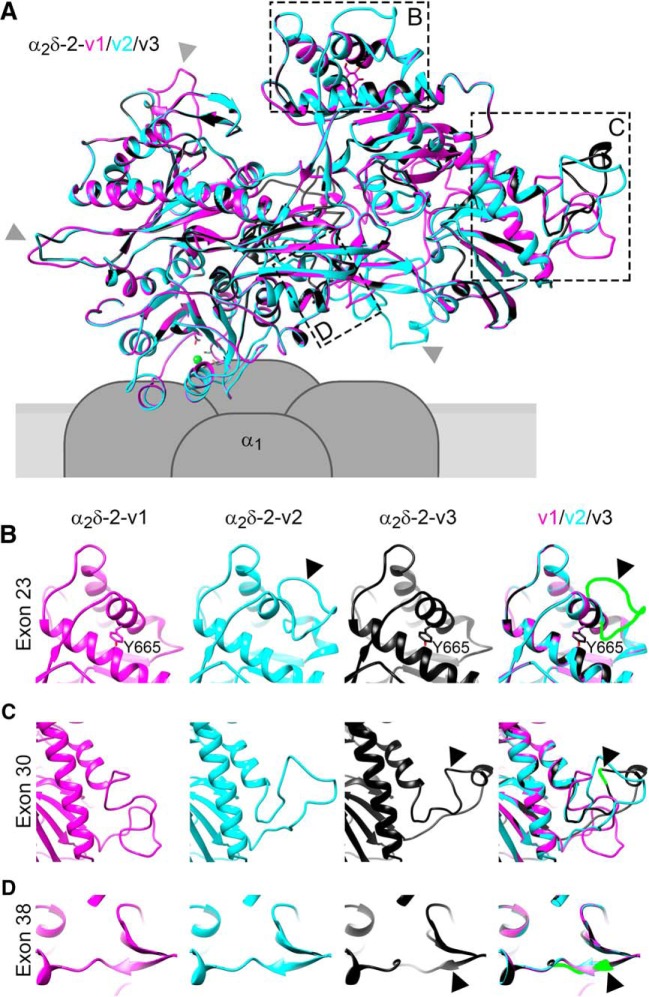
Splicing sites of α_2_δ-2 and implications on secondary structure. ***A***, Using homology modeling, we tested the potential consequences of alternative splicing on the structure prediction of three distinct α_2_δ-2 splice variants (v1–v3) based on the high-resolution structure of α_2_δ-1 (PDB code: 5GJV; Wu et al., 2016). Although some of the longer loops generally seemed to be quite flexible and differently orientated in all three models (gray arrows), alternative splicing of exons 23, 30, and 38 resulted in differences between secondary structure elements in distinct variants (boxed regions in ***B***–***D***). ***B***, Higher magnification showing that the inclusion of exon 23 in α_2_δ-2-v2 suggests the formation of an extra loop (arrowhead and green selection in overlay), leading to the disruption of an α-helix present in α_2_δ-2-v1 and α_2_δ-2-v3 (see also Fig. 13). ***C***, Structure modeling further proposes that the insertion of three base pairs in exon 30 of α_2_δ-2-v3 causes the formation of a short α-helix in the depicted loop, which is absent in α_2_δ-2-v1 and α_2_δ-2-v2. ***D***, Moreover, models implicate that alternative splicing of exon 38 affects the length of a β sheet at this position, which is longer in α_2_δ-2-v1 and α_2_δ-2-v2.

Our results revealed a specific trans-synaptic function of only one of the three expressed α_2_δ isoforms, namely α_2_δ-2. Sequence comparisons of the previously known alternatively spliced regions (Hobom et al., 2000) identified three potential splice sites in α_2_δ-2 (Fig. 13*A*, scheme): inclusion or absence of exon 23 and two alternative splice sites at exons 30 and 38. Using homology modeling, we explored the potential implication of these sequence differences on the structural fold of α_2_δ-2 (Fig. 14) based on the recently suggested α_2_δ-1 cryo-EM structure (Wu et al., 2016). Inclusion of exon 23 resulted in the formation of an extra loop that might disrupt an α-helix (Figs. 13*B*, 14*B*), whereas alternative splicing of exons 30 and 38 predicted only small changes in the secondary structure (Fig. 14*C*,*D*). Therefore, to test whether alternative splicing at these sites is involved in the trans-synaptic function of α_2_δ-2, we cloned and compared three splicing variants (α_2_δ-2-v1, α_2_δ-2-v2, and α_2_δ-2-v3, α_2_δ-2-v1 = original construct; Fig. 13*A*). To determine the potential of the splice variants in inducing mismatched synapses, we transfected WT hippocampal neurons with the three distinct α_2_δ-2 variants together with soluble eGFP. Strikingly, only the splice variants lacking exon 23 (α_2_δ-2-v1 and α_2_δ-2-v3) induced the clustering of postsynaptic GABA_A_Rs opposite transfected glutamatergic nerve terminals (Fig. 13*C*, ΔExon 23). The fact that α_2_δ-2-v1 and α_2_δ-2-v3 both induced mismatched synapse formation excludes a major role of exons 30 and 38, which differ in α_2_δ-2-v1 and α_2_δ-2-v3. In contrast, α_2_δ-2-v2, which contains the exon 23 insert, failed completely to induce mismatched synapse formation (Fig. 13*C*, +Exon 23), indicating that the trans-synaptic function of α_2_δ-2 on GABA_A_Rs is transcriptionally regulated. Together, our results have uncovered a highly specific role of the α_2_δ-2 subunit of voltage-gated calcium channels in clustering postsynaptic GABA_A_Rs, an important process that is further regulated by the presence of α-Nrxns and by alternative splicing.

## Discussion

The experiments described here reveal that presynaptic expression of a single splice variant of α_2_δ-2 trans-synaptically regulates postsynaptic GABA_A_R abundance. Therefore, presynaptic α_2_δ-2 modulates postsynaptic GABA_A_R clustering independently of the presynaptic neurotransmitter, induces an aberrant wiring of glutamatergic axons with postsynaptic GABAergic sites of both glutamatergic and GABAergic neurons, and alters synaptic transmission in mismatched synaptic pairs. These results demonstrate for the first time that presynaptic α_2_δ subunits are able to trans-synaptically regulate synapse specification and axonal wiring.

### α_2_δ-2 induces the formation of mismatched synapses via a trans-synaptic mechanism

Excitatory and inhibitory synapses are characterized by their distinct molecular composition and subcellular localization in neurons. The proper alignment of presynaptic and postsynaptic specializations is a hallmark of synapse formation and a prerequisite for efficient and synchronous excitatory and inhibitory synaptic transmission (Lardi-Studler and Fritschy, 2007). Only a small number of studies have reported mismatched synapses *in vitro*. In micro-island cultures of autaptic hippocampal neurons, the vast majority of GABA_A_Rs and gephyrin clusters are located opposite glutamatergic terminals, suggesting that postsynaptic compartments can be recruited without the proper presynaptic input (Rao et al., 2000; Christie et al., 2002). Moreover, in low-density cultures of hippocampal and cerebellar granule neurons, mismatches of presynaptic and postsynaptic proteins can form during early developmental stages, but are essentially absent in differentiated cultures (Brünig et al., 2002; Anderson et al., 2004; Studler et al., 2005). In contrast to these studies, the mismatched synapses reported in our study are specifically induced by the presynaptic expression of α_2_δ-2 and are independent from neurotransmitter input, suggesting that α_2_δ-2 operates via a different mechanism.

Mixed synapses with glutamatergic and GABAergic properties have also been detected in different brain areas, indicating a functional role *in vivo*. For example, postsynaptic GABA_A_Rs are present opposite presynaptic glutamatergic mossy fiber axon terminals in the cerebellar cortex and hippocampus (Nusser et al., 1996a,b; Bergersen et al., 2003). Physiologically, these receptors may be involved in fine-tuning excitatory synaptic transmission in response to GABA spillover from surrounding synapses (Isaacson et al., 1993). Although we cannot completely rule out GABA spillover from adjacent neurons, it is important to note that the α_2_δ-2-induced mismatched synapses do not express presynaptic vGAT. Therefore, the recruitment of postsynaptic GABA_A_Rs is entirely independent of presynaptic GABA release. These findings are consistent with a recent report showing that, even in the absence of presynaptic glutamate release, dendritic spines are formed and AMPARs are present in glutamatergic synapses (Sigler et al., 2017).

Generally, excitatory and inhibitory synapses are mostly targeted to different subcellular regions of the postsynaptic neuron (Lardi-Studler and Fritschy, 2007). Therefore, glutamatergic boutons predominantly contact postsynaptic dendritic spines (Peters, 2002), whereas GABAergic nerve terminals mostly connect to the shaft of the dendrite (Markram et al., 2004). Nevertheless, converging lines of evidence demonstrate morphologically mixed synapse types in different parts of the brain. In the adult neocortex, for example, two distinct types of GABAergic synapses are positioned either on the shaft or spine of the dendrite, which are suggested to have different roles in shaping dendritic activity (Chen et al., 2012). It is hypothesized that these constellations facilitate the shunting of excitatory currents, thereby balancing excitatory and inhibitory neurotransmission (Nusser et al., 1996a; Root et al., 2014). Along these lines, we proposed that the underlying mechanism of α_2_δ-2-induced mismatched synapses could be explained by a compensatory upregulation of postsynaptic GABA_A_Rs due to increased excitatory transmission upon α_2_δ overexpression (Zhou and Luo, 2015; Chen et al., 2018). However, several of our observations argue against this theory. First, using distinct experimental paradigms, we show here that α_2_δ-1 and α_2_δ-3, which both enhance excitatory synaptic transmission and increase presynaptic release probability (Hoppa et al., 2012), do not induce postsynaptic clustering of synaptic GABA_A_R subtypes. Second, the effect of α_2_δ-2 is independent of the presynaptic neurotransmitter identity, thus providing compelling evidence for a trans-synaptic function. Third, solely α_2_δ-2 splice variants lacking exon 23 specifically induced the recruitment of postsynaptic GABA_A_Rs opposite glutamatergic nerve terminals. Fourth, the effect of α_2_δ-2 is restricted to transfected synapses, emphasizing a synapse-specific role rather than a global alteration of GABAergic postsynaptic components.

### Presynaptic α_2_δ subunits differentially modulate postsynaptic GABA_A_R abundance via distinct mechanisms

We found diverging effects of presynaptic α_2_δ-2 and α_2_δ-3 on the postsynaptic GABA_A_R abundance of GABAergic MSNs because α_2_δ-2 strongly increased and α_2_δ-3 reduced postsynaptic GABA_A_R levels (Fig. 8). In contrast to presynaptic α_2_δ-2 expression, which modulated the postsynaptic composition without apparently affecting the presynaptic side, the effect of α_2_δ-3 was associated with smaller presynaptic boutons and decreased presynaptic vGAT content. Smaller presynaptic boutons may also explain the reduced GLUR1 content opposite α_2_δ-3-expressing synapses (Fig. 2). Together, these data suggest that presynaptic α_2_δ subunits regulate presynaptic differentiation and postsynaptic GABA_A_R abundance by two independent mechanisms. Although expression of α_2_δ-3 induces smaller synapses associated with a reduced postsynaptic receptor density, presynaptic α_2_δ-2 modulates postsynaptic GABA_A_R abundance but does not affect presynaptic bouton size. Altered expression of mammalian α_2_δ-3 (Pirone et al., 2014) and its invertebrate homologs (Kurshan et al., 2009; Caylor et al., 2013) has previously been shown to affect the size and morphology of presynaptic boutons of auditory nerve fibers and motoneurons, respectively.

### Evidence for an α_2_δ-2-mediated trans-synaptic signaling cascade

To date, surprisingly little is known on the specific signals imperative for the proper alignment of inhibitory synapses and the targeting of GABA_A_Rs and gephyrin to the postsynaptic compartment. *In vitro* cultured neurons provide an excellent and reduced model system allowing us to address basic cell biological mechanisms. Most importantly, they are less prone to potential compensatory mechanisms compared with the *in vivo* situation (Dong et al., 2007; Brown et al., 2016; Chen et al., 2017). Likewise, overexpression of proteins in their native environment provides a powerful experimental tool but needs to be carefully discussed, particularly in relation to the specific physiological roles. Therefore, it is conceivable that an aberrantly high accumulation of presynaptic α_2_δ-2 proteins may induce artificial synaptic connections. Nevertheless, our study provides several lines of evidence supporting a presynaptic role of α_2_δ-2 in the trans-synaptic alignment of synapses and receptor clustering. First, we observed that presynaptic expression of a single splice variant of α_2_δ-2 trans-synaptically regulates postsynaptic GABA_A_R abundance. Second, overexpression of α_2_δ-2 specifically induces an aberrant wiring of glutamatergic axons to postsynaptic GABAergic sites independently of the postsynaptic neuron type (Fig. 11). Third, whereas mismatched synapses lack glutamate receptors, they contain different synaptic GABA_A_R subtypes and gephyrin. Importantly, the strongly reduced GLUR1 expression (Fig. 2) in the mismatched and aberrantly wired synapses is a secondary effect because no AMPARs are to be expected in postsynaptic GABAergic synapses (Fig. 5*C*, model). Fourth, gephyrin abundance is higher in α_2_δ-2-induced mismatched synapses compared with untransfected endogenous GABAergic synapses. Considering that gephyrin immobilizes synaptic GABA_A_R clusters in hippocampal neurons (Jacob et al., 2005), we provide strong evidence that gephyrin recruitment occurs secondary to synaptic GABA_A_R clustering.

However, it is still unknown whether the interaction with postsynaptic receptors is mediated by a direct trans-synaptic action of α_2_δ-2 or if it may be indirectly mediated by the interaction with other primary synaptic organizers (see also discussion in Fell et al., 2016). Several studies implicate the trans-synaptic cell adhesion molecules α-Nrxns as the most interesting candidates for mediating a possible indirect interaction between α_2_δ-2 and GABA_A_Rs (Missler et al., 2003; Kang et al., 2008; Born et al., 2014; Tong et al., 2017). Here, we demonstrate that presynaptic α_2_δ-2 also potently induces mismatched synapse formation in the absence of α-Nrxns. Although this finding supports an α_2_δ-2-mediated trans-synaptic effect and possibly a direct trans-synaptic interaction of α_2_δ-2 with postsynaptic GABA_A_Rs, it does not exclude the potential involvement of other cell adhesion molecules not addressed in our study (Graf et al., 2004; Lardi-Studler and Fritschy, 2007).

The effect of overexpressed α_2_δ-2 was more pronounced in α-Nrxn TKO synapses compared with WT control synapses. We observed that the baseline expression of postsynaptic GABA_A_Rs in TKO was already significantly increased, which shows that no overexpression of α_2_δ-2 is needed for inducing postsynaptic GABA_A_R clustering. This finding may indicate that the presence of α-Nrxns negatively influences GABA_A_R recruitment by α_2_δ-2. Alternatively, the loss of α-Nrxns could be either overproportionally compensated by the still present β-neurexins (Missler et al., 2003) or shift the modulation of postsynaptic receptor abundance to the neuroligin-2/Slitrk3 or the neuroligin-2/MDGA1 complexes (Pettem et al., 2013; Li et al., 2017). Independently of the detailed underlying mechanism, our findings support a cooperative action of α_2_δ subunits and neurexins in fine-tuning synaptic functions (Tong et al., 2017; Brockhaus et al., 2018).

### Future implications and potential relevance to neurological disorders

One important finding presented here is that only α_2_δ-2 splice variants lacking exon 23 induced a mismatched localization of postsynaptic GABA_A_Rs. This is interesting for several reasons. First, the presynaptic effect of α_2_δ-2 lacking a single spliced exon is highly specific and thus argues against a simple unspecific effect caused by homologous overexpression of α_2_δ isoforms in our experimental paradigm. Second, in light of discussing potential interaction mechanisms (see above), it is tempting to speculate that α_2_δ-2 interacts with its partners via the region surrounding the lacking exon 23, which will be tested in future experiments. Third, our findings show that the trans-synaptic function of α_2_δ-2 may be independent of the calcium channel complex. This is further supported by a previous study showing that heterologous coexpression of different α_2_δ-2 splice variants with various α_1_ subunits caused similar effects on calcium channel current densities and activation/inactivation kinetics (Hobom et al., 2000). Fourth, and most importantly, alternative splicing has also been described for mouse and human genes encoding for α_2_δ-1 and α_2_δ-4 (Lana et al., 2014; Bacchi et al., 2015). This is particularly interesting considering the previously identified associations between α_2_δ subunits with neurological disorders (Chioza et al., 2009; Edvardson et al., 2013; Pippucci et al., 2013; Vergult et al., 2015; Butler et al., 2018; Valence et al., 2018). Two distinct splice site mutations in CACNA2D2, for example, lead to the truncation of the protein, causing epilepsy, dyskinesia, and cerebellar atrophy in one patient (Pippucci et al., 2013), whereas the other mutation is associated with congenital ataxia (Valence et al., 2018). These clinically observed phenotypes are similar to the previously described phenotype of Cacna2d2 mutant or null mouse models (Barclay et al., 2001; Brodbeck et al., 2002; Ivanov et al., 2004) and may be caused by the loss of functional GABAergic synapses and the consequential increased excitatory activity. Moreover, α_2_δ subunits are potential risk genes for autism spectrum disorders (Iossifov et al., 2012; De Rubeis et al., 2014) and schizophrenia (Purcell et al., 2014; Moons et al., 2016), diseases classically associated with altered neuronal connectivity (Belmonte et al., 2004; Fornito and Bullmore, 2015). The findings presented here provide a possible mechanistic explanation for how altered α_2_δ subunit expression may be linked to neurological disorders. However, for the future understanding of the precise underlying disease mechanisms, it will be necessary to reveal whether all α_2_δ subunit isoforms can act as similarly specific trans-synaptic organizers in distinct synapses. Finally, our findings allow hypothesizing that presynaptic α_2_δ subunits are critical determinants of synapse specificity and connectivity. Therefore, alterations in the balance of presynaptic α_2_δ isoforms may underlie synaptic plasticity and, conversely, pathological axonal wiring observed in neurological disorders.
